# Transit through the Flea Vector Induces a Pretransmission Innate Immunity Resistance Phenotype in *Yersinia pestis*

**DOI:** 10.1371/journal.ppat.1000783

**Published:** 2010-02-26

**Authors:** Viveka Vadyvaloo, Clayton Jarrett, Daniel E. Sturdevant, Florent Sebbane, B. Joseph Hinnebusch

**Affiliations:** 1 Laboratory of Zoonotic Pathogens, Rocky Mountain Laboratories, National Institute of Allergy and Infectious Diseases, National Institutes of Health, Hamilton, Montana, United States of America; 2 Genomics Unit, Research Technologies Section; Rocky Mountain Laboratories, National Institute of Allergy and Infectious Diseases, National Institutes of Health, Hamilton, Montana, United States of America; 3 Institut National de la Santé et de la Recherche Médicale U801, Institut Pasteur de Lille, and Université Lille Nord de France, Lille, France; University of Toronto, Canada

## Abstract

*Yersinia pestis*, the agent of plague, is transmitted to mammals by infected fleas. *Y. pestis* exhibits a distinct life stage in the flea, where it grows in the form of a cohesive biofilm that promotes transmission. After transmission, the temperature shift to 37°C induces many known virulence factors of *Y. pestis* that confer resistance to innate immunity. These factors are not produced in the low-temperature environment of the flea, however, suggesting that *Y. pestis* is vulnerable to the initial encounter with innate immune cells at the flea bite site. In this study, we used whole-genome microarrays to compare the *Y. pestis in vivo* transcriptome in infective fleas to *in vitro* transcriptomes in temperature-matched biofilm and planktonic cultures, and to the previously characterized *in vivo* gene expression profile in the rat bubo. In addition to genes involved in metabolic adaptation to the flea gut and biofilm formation, several genes with known or predicted roles in resistance to innate immunity and pathogenicity in the mammal were upregulated in the flea. *Y. pestis* from infected fleas were more resistant to phagocytosis by macrophages than *in vitro*-grown bacteria, in part attributable to a cluster of insecticidal-like toxin genes that were highly expressed only in the flea. Our results suggest that transit through the flea vector induces a phenotype that enhances survival and dissemination of *Y. pestis* after transmission to the mammalian host.

## Author Summary

Bubonic plague cycles depend on the ability of *Yersinia pestis* to alternately infect two very different hosts—a mammal and a flea. Like any arthropod-borne pathogen, *Y. pestis* must sense host-specific environmental cues and regulate gene expression accordingly to produce a transmissible infection in the flea after being taken up in a blood meal, and again when it exits the flea and enters the mammal. We examined the *Y. pestis* phenotype at the point of transmission by *in vivo* gene expression analyses, the first description of the transcriptome of an arthropod-borne bacterium in its vector. In addition to genes associated with physiological adaptation to the flea gut, several *Y. pestis* virulence factors required for resistance to innate immunity and dissemination in the mammal were induced in the flea, suggesting that the arthropod life stage primes *Y. pestis* for successful infection of the mammal.

## Introduction

Arthropod-borne transmission of bacterial pathogens is somewhat rare but has evolved in a phylogenetically diverse group that includes the rickettsiae, *Borrelia* spirochetes, and the gram-negative bacteria *Francisella tularensis* and *Yersinia pestis*, the plague bacillus. *Y. pestis* circulates among many species of wild rodents, its primary reservoir hosts, via flea bite. As it alternates between fleas and mammals, it is postulated that *Y. pestis* regulates gene expression appropriately to adapt to the two disparate host environments, and that different sets of genes are required to produce a transmissible infection in the flea and disease in the mammal.

Many important *Y. pestis* virulence factors that are required for plague in mammals have been identified, and most of them are induced by a temperature shift from <26°C to 37°C, which mimics the transition from a flea to the warm-blooded host [1]. To date, only three transmission factors (genes specifically required to produce a transmissible infection in the flea) have been characterized. One, the yersinia murine toxin (*ymt*) gene, encodes a phospholipase D that is required for survival in the flea midgut [2]. The other two, (*hmsHFRS* and *gmhA*), are responsible for an extracellular polysaccharide and a lipopolysaccharide (LPS) core modification that are required for normal biofilm formation and blockage in the flea [3],[4]. Biofilm development in the flea digestive tract is important for biological transmission [5],[6],[7]. After being taken up in a blood meal, *Y. pestis* proliferates in the lumen of the flea midgut to form cohesive multicellular biofilm aggregates. In some infected fleas, the proventricular valve between the midgut and esophagus is colonized. The subsequent growth and consolidation of the adherent *Y. pestis* biofilm amongst the rows of cuticle-covered spines that line the proventriculus interferes with normal blood feeding, resulting in regurgitation of bacteria and transmission. Fleas with a completely blocked proventriculus make prolonged, repeated attempts to feed, increasing the opportunities for transmission.

Formation of a *Y. pestis* biofilm *in vitro* and in the flea proventriculus depends on synthesis of an extracellular polysaccharide matrix (ECM) that is synthesized only at temperatures below 26°C [3],[7]. In common with many other bacteria, ECM synthesis in *Y. pestis* is controlled by intracellular levels of cyclic di-GMP, which are determined by competing activities of the *hmsT* diguanylate cyclase and *hmsP* phosphodiesterase gene products [8],[9]. Bacterial adhesins are typically required for initial adherence and autoaggregation in biofilm development [10], but such factors have yet to be identified in *Y. pestis*.

In a previous study, we reported the *in vivo* gene expression profile of *Y. pestis* during bubonic plague in rats [11]. In this study, we characterized the *Y. pestis* transcriptome in blocked *Xenopsylla cheopis* rat fleas, an important vector of plague to humans. Comparing the *Y. pestis* gene expression profile in the flea to those of *in vitro* biofilm and planktonic cells cultured at the low temperature typical of the flea implicated several genes in a flea-specific adaptive response and in proventricular blockage. In addition, comparing the gene expression patterns in the flea and in the rat bubo confirmed that distinct subsets of genes are differentially expressed during the *Y. pestis* life cycle. Notably, several genes with known or predicted roles in protection against the mammalian innate immune system and in pathogenesis were upregulated in the flea, suggesting that transit through the insect vector preinduces a phenotype that enhances *Y. pestis* survival and dissemination in the mammal after flea-borne transmission.

## Results/Discussion

### Transcriptional profile of *Y. pestis* in the flea

Little is known about the environmental conditions in the flea digestive tract, how *Y. pestis* adapts to them, or the physiological state of the bacteria at transmission when they exit the flea and enter the mammal. Adult fleas are obligate blood feeders and take frequent blood meals, consisting primarily of protein and lipid with relatively little carbohydrate. Flea proteases, lipases, and other digestive enzymes begin to process the blood meal in the midgut immediately after feeding, yielding amino acids and peptides, glycerol, fatty acids, and simple carbohydrates [12]. This provides the “medium” for *Y. pestis* growth, but these and other factors such as pH, oxygen tension, osmolarity, and flea antibacterial immune components are poorly defined. During the first week after being ingested in an infectious blood meal, *Y. pestis* grows rapidly in the flea midgut to form large bacterial aggregates. Bacterial load peaks at about 10^6^ cells per flea as the *Y. pestis* biofilm accumulates in the proventriculus to cause blockage, and then plateaus [2],[3].

In this study, we determined the *Y. pestis* gene expression profile in infective, blocked fleas, in which the proventriculus was occluded with a mature bacterial biofilm. *Y. pestis* KIM6+, which lacks the 70-kb virulence plasmid that is not required for flea infection or blockage [3] was used for this analysis. Blockage occurred between 1.5 and 3.5 weeks after the initial infectious blood meal, during which time the fleas fed on uninfected mice twice weekly. The *Y. pestis in vivo* biofilm transcriptome was compared to the transcriptomes of *in vitro* biofilm and planktonic cultures grown at 21°C, the same temperature at which the fleas were maintained.

**Figure 1:**
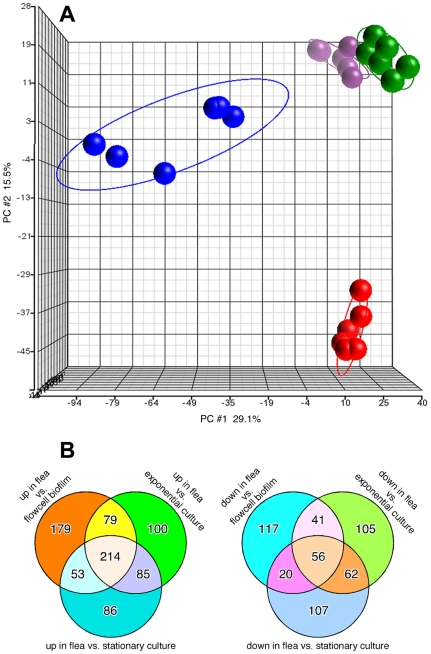
Distinct transcriptional profile of *Y. pestis* in infected fleas. (A) Principal Component Analysis (PCA) representation of replicate microarray gene expression profiles of *Y. pestis* KIM6+ from blocked fleas (blue symbols) and from *in vitro* flowcells, exponential phase planktonic cultures, and stationary phase planktonic cultures (red, green, and purple symbols, respectively). (B) Venn diagrams representing the number of *Y. pestis* genes upregulated or downregulated ≥2-fold in the flea relative to *in vitro* culture conditions.

Expression of 55% of *Y. pestis* ORFs was detected in the flea samples; and 74 to 79% in the *in vitro* biofilm, exponential phase planktonic and stationary phase planktonic cultures. Principal component analysis to visualize overall clustering of the microarray data showed that the transcriptional profiles were reproducible and discrete for the *in vitro* and *in vivo* conditions (Fig. 1A). Profiles of the exponential and stationary phase planktonic cultures clustered most closely, whereas the profiles from *in vitro* and *in vivo* biofilm growth were more distinct from each other and from the planktonic culture profiles. There were 214 *Y. pestis* genes whose expression was significantly upregulated and 56 genes downregulated in the flea compared to all *in vitro* growth conditions (Fig. 1B; Tables S1 and S2). Quantitative RT-PCR analysis of a subset of *Y. pestis* genes differentially expressed in the flea was confirmatory of the microarray results (Fig. S2).

### *Y. pestis* metabolic adaptation to the flea gut environment

**Figure 2:**
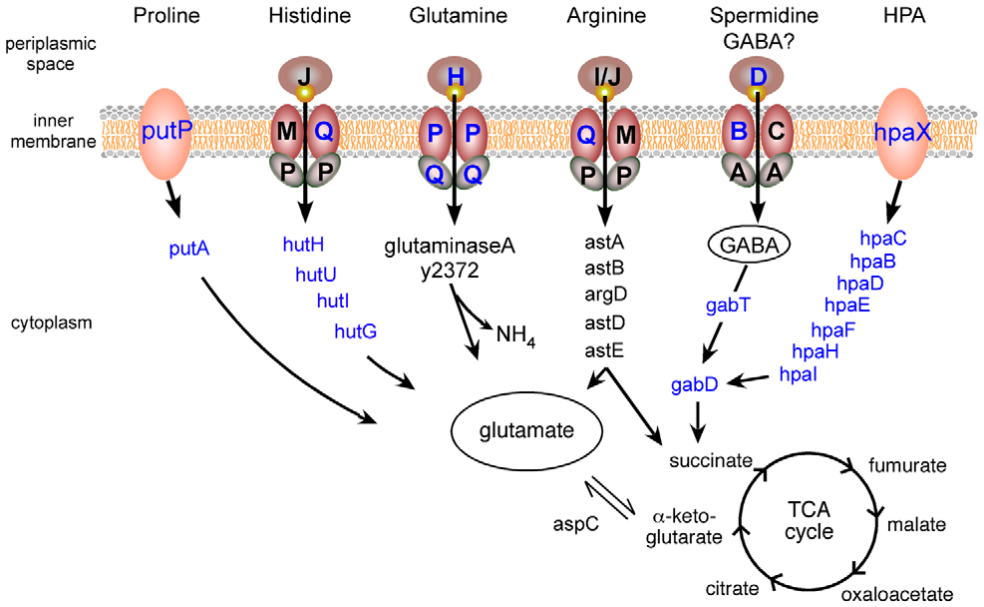
*Y. pestis* amino acid uptake and catabolism pathways upregulated in the flea. Periplasmic and inner membrane uptake proteins for proline (PutP), histidine (HisJMPQ), glutamine (GlnHPQ), arginine (ArtIMPQ), spermidine (PotABCD), and hydroxyphenylacetate (HpaX) are indicated from left to right. Genes encoding catabolic enzymes leading to glutamate and TCA cycle intermediates are also shown. Symbols labeled in blue indicate genes upregulated ≥2-fold in the flea compared to all *in vitro* conditions (Table S1).

Of the 214 genes upregulated in the flea gut compared to all *in vitro* conditions, 78 are metabolic genes, 60 of which are involved in uptake and catabolism of amino acids and carbohydrates (Table S1). In particular, genes involved in transport and catabolism of the L-glutamate group of amino acids (Gln, His, Arg, and Pro) were specifically upregulated in the flea (Fig. 2). The degradation of these amino acids gives rise to L-glutamate and the TCA cycle intermediates succinate, formate, and α-ketoglutarate. The *gabD* and *gabT* genes involved in the production of succinate from γ-aminobutyrate (GABA), another member of the L-glutamate group, were also highly induced in the flea. The *gabD* gene functions to produce succinate from both GABA and hydroxyphenylacetate (HPA), an aromatic degradation product of Tyr and Phe; and the HPA transport (*hpaX*) and catabolism (*hpaCBIFHDE*) genes of *Y. pestis* were also highly upregulated in the flea gut (Table S1, Fig. 2). As *Y. pestis* does not have homologs of genes required to produce GABA or HPA, these metabolites may be taken up from the flea digestive tract. Alternatively, the *gabD* and *gabT* gene products might act in the reverse direction to synthesize GABA, which has osmoprotective properties [13]. The central role of the L-glutamate family of amino acids may also confer this advantage in the flea gut, because Glu and Pro are osmoprotectants. Interestingly, both glutamate and GABA are important neurotransmitters at the neuromuscular junction of insects, and the concentration of glutamate is very low in insect hemolymph, suggesting that it is converted to glutamine before it is absorbed [14]. Insect midgut epithelium is typified by multiple amino acid transporters with specific substrates and rapid absorption kinetics, but different amino acids enter the hemocoel at different rates and amounts [14],[15]. Thus, *Y. pestis* metabolism in the flea may reflect the available pool of amino acids in the midgut.

In contrast to the amino acids, hexoses do not appear to be an important energy source during infection of the flea. Only the genes encoding for chitobiose phosphotransferase (PTS) uptake and utilization systems (*chbBC*; *chbF*), and for a PTS system of unknown specificity (*frwBCD*) were significantly upregulated in the flea [16],[17]. Chitobiose could be present in the flea gut due to turnover of the chitin layer on the proventricular spines. Expression of the glucose PTS system was only slightly increased relative to LB cultures, and other PTS systems were downregulated (Table S2). Glycolytic pathways were not upregulated in the flea; instead, available hexoses and the gluconeogenesis pathway may be used to synthesize polysaccharide components required for cell growth. Upregulation of the *actP* and *acs* genes in the flea, which direct the uptake of acetate and its conversion to acetyl-CoA, also suggests that insufficient acetyl-CoA is produced by glycolysis to potentiate the TCA cycle. The switch from acetate secretion to acetate uptake is typical of growth in a glucose-limited, amino acid rich environment [18]. In contrast to hexose uptake systems, *Y. pestis* genes that encode permeases for the pentoses ribose, xylose, and arabinose were induced in the flea gut. Acquisition of pentoses from the environment may be important because *Y. pestis* does not possess glucose 6-phosphate dehydrogenase activity, the first step of the pentose phosphate pathway [19].

Although the flea gut contains lipid derived from the blood meal, *Y. pestis* does not appear to use it as a major energy source. None of the fatty acid uptake or catabolism genes were upregulated in the flea compared to growth in LB. However, genes for glycerol and glycerol-3-phosphate uptake and utilization were upregulated, suggesting that flea digestion products derived from blood glycerolipids may be used by *Y. pestis*. In summary, *Y. pestis* appears to use amino acids, particularly the L-glutamate family, as primary carbon, nitrogen, and energy sources in the flea. Amino acid carbon is presumably funneled into the TCA cycle, the genes for which are highly expressed in the flea (Table S3).

### *Y. pestis* genes involved in infection and biofilm formation in the flea

Because blockage of the flea vector is essentially a biofilm phenomenon, *Y. pestis* genes whose expression patterns are significantly upregulated in the flea and flowcell biofilms relative to planktonic cultures (Table S4) might indicate that they are transmission factors. Several studies comparing the transcriptional profiles of *Escherichia coli* and other gram negative bacteria during biofilm and planktonic growth *in vitro* have been published [20],[21],[22],[23]. Certain genes whose mutational loss resulted in an altered biofilm phenotype have been identified in these studies; but in general a consistent, distinct biofilm gene expression profile has not emerged. This is probably because different media and experimental systems have been employed and the fact that a biofilm consists of a physiologically heterogeneous community [24],[25]. Nevertheless, common biofilm-related adaptations include the repression of motility and the induction of specific adhesins, an extracellular polysaccharide matrix (ECM), and an envelope stress response (ESR) [10],[23]. However, *Y. pestis* is constitutively nonmotile, and synthesis of the Hms-dependent biofilm ECM is regulated post-translationally [26]. The *ymt* gene was among the most highly expressed genes in the flea (Table S3), but neither it nor the known transmission factors (*hmsHFRS*, *hmsT*, *hmsP*, and *gmhA*) showed significantly higher expression in the flea than *in vitro* at 21°C, indicating that they are induced primarily by low temperature, and not by environmental factors specific to the flea gut. *Y. pestis* homologs of two genes with previously identified roles in biofilm, *yidE*, which encodes a hyperadherence factor in *E. coli* [27], and *cpxP*, a member of the *cpxPAR* ESR system, were upregulated in the flowcell; but predicted adhesin genes were not upregulated.

The transcriptional profile of *Y. pestis* in blocked fleas showed greater similarity to the transcriptional profile reported for *E. coli* in mature, four-day-old *in vitro* biofilms [23]. In addition to *yidE* and *cpxP*, other *Y. pestis* predicted adhesins and components of an ESR were upregulated in the flea. The *Y. pestis* homologs of *Pseudomonas aeruginosa cupA1* and *cupA3* in a predicted fimbrial biosynthesis operon and *yapL*, a predicted autotransporter adhesin similar to *E. coli tibA*, were specifically upregulated in the flea (Table S1). The *cupA* fimbrial locus and *tibA* are important for surface adherence and for biofilm formation in *P. aeruginosa* and *E. coli*, respectively [28],[29]. Evidence for induction of an ESR in the flea included the high expression levels of *rpoE*, the gene for the alternate transcription factor σ^E^ (as well as the anti-σ^E^ negative regulator genes *rseA* and *rseB*), *cpxP*; and *pspA* and *pspG*, components of the phage-shock protein (Psp) response (Tables S1 and S3). These genes were also found to be upregulated in mature *E. coli* biofilms [23], suggesting that the three prominent ESR systems are important for integrating signals required for survival in a biofilm.

Because homologs of the *yidE*, *cpxP*, *tibA (yapL)*, *cupA* fimbriae, and *pspABC* genes were upregulated in the flea and have been shown to be involved in biofilm formation in other bacteria [23],[27],[28],[29], we made a series of *Y. pestis* strains containing deletions of these loci. However, the single loss of any of these genes did not result in a noticeable defect in biofilm formation *in vitro*, or in flea infection or blockage (data not shown). These genes may contribute to biofilm formation, but are not individually essential for this phenotype. Although genes in the polyamine transport *gabTpotDBC* locus are among the most highly induced genes in the flea (Table S1) and polyamines are essential for *Y. pestis* biofilm formation [30], we have previously reported that a *Y. pestis* Δ*pot* mutant has no defect in flea infection or blockage [31]. This is likely due to the fact that *Y. pestis* is able to synthesize polyamines *de novo*.

### Differential gene expression during the *Y. pestis* life cycle

**Figure 3:**
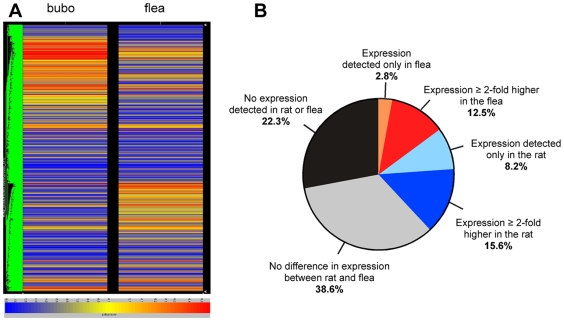
Distinct *Y. pestis* gene expression profiles in flea and rat hosts. (A) Hierarchichal clustering of normalized microarray data sets of *Y. pestis* gene expression in the rat bubo and the flea. The scale indicates relative transcript levels (blue = low; red = high) for all 4,638 *Y. pestis* genes on the microarray. (B) Percentages of *Y. pestis* genes that are differentially regulated (or not) in the flea and in the rat bubo.

With this study, the *in vivo* transcriptome of *Y. pestis* in blocked fleas and in the rat bubo [11] have now both been characterized. A comparison of normalized gene expression levels from the two data sets provides insight into the biology of the flea-mammal life cycle. About 15% of *Y. pestis* genes showed significantly higher relative expression levels or expression only in the flea than in the bubo; 24% were more highly expressed in the bubo than in the flea; and 61% were not differentially expressed in the two hosts (Fig. 3).

Several virulence factors were differentially regulated in the two hosts, but others were not (Table 1). In addition to the known temperature-induced virulence factors, iron acquisition systems, including the *ybt* and *yfe* operons that are required for virulence; and oxidative and nitrosative stress response genes, including the *hmp* virulence factor, are highly upregulated in the rat bubo, but not the flea. The analysis also reinforces the model that *Y. pestis* produces a hexaacylated lipid A in the flea, and that the change to the less immunostimulatory tetraacylated form occurs only after transmission [32]. Other virulence and transmission factors were not differentially regulated, including the *hms* genes; and the *Y. pestis* plasminogen activator (*pla*), critical for dissemination from extravascular tissue at the fleabite site [33], and *ymt* were highly expressed in both hosts (Table S3 and [11]). The *Y. pestis* outer surface protein gene *yadB*, recently shown to be required for dissemination and bubonic plague pathogenesis from a subcutaneous inoculation site [34], was significantly upregulated in both the flea and the bubo compared to *in vitro* conditions (Tables 1, S1).

**Table 1:** Differential expression of *Y. pestis* pathogenesis-related genes in the flea.

Gene or system	Description/function	Expression level in flea relative to*:			
		Flow cell	Exp. phase	Stat. phase	Bubo
***Outer surface components***					
*caf1R*	F1 capsule gene regulator	*f*	*f*	*f*	−2.4
*caf1M*	F1 capsule periplasmic chaperone	*f*	*f*	*f*	−20
*caf1A*	F1 capsule outer membrane usher	7.1	5.3	4.0	−36
*caf1*	F1 capsule subunit protein	6.8	5.0	4.5	−42
*pla*	plasminogen activator	0.9	1.0	0.8	−1.5
*yadB*	YadA-like protein	*f*	*f*	*f*	1.1
*yadC*	YadA-like protein	2.7	3.3	1.9	*f*
*psaE*	pH 6 antigen regulator	−4.4	−7.9	−20	−12.6
*psaF*	pH 6 antigen hypothetical protein	1.2	1.0	−2.7	−0.8
*psaA*	pH 6 antigen fimbrial subunit	*ns*	*ns*	*ns*	*b*
*psaB*	pH 6 antigen chaperone	*ns*	*ns*	*ns*	*ns*
*psaC*	pH 6 antigen usher	3.4	3.4	2.5	−0.6
*lpxP*	hexaacylated lipid A synthesis	2.2	3.4	3.2	1.8
*msbB*	hexaacylated lipid A synthesis	1.5	1.6	1.4	2.2
***Iron acquisition systems***					
Ybt	yersiniabactin siderophore, 10 genes	1.5–4.2	−1.4–1.8	−4.6–1.0	−15–−84
Yfe	ABC iron transporter, 5 genes	−1.4–1.8	−1.6–1.3	−1.4–1.6	−1.2–−14
***Toxins***					
*yitR*	positive regulator of *yitABC*	10.3	47	23	*f*
*yitABC*	insecticidal-like toxin complex	5.6–11	21–27	8.8–10	17–25
*yipB*	insecticidal-like toxin complex	4.0	10	5.6	2.2
***Global regulatory systems***					
*rovA*	regulator of virulence factors	−7.3	−6.6	−5.9	−1.4
*rovM*	repressor of *rovA*	9.1	7.1	5.9	11.1
*phoP*	regulator of PhoPQ regulon	2.1	2.2	2.2	2.3
***Other***					
*mgtC*	survival in macrophages	3.0	5.9	4.3	10.0
*mviM*	*Salmonella* mouse virulence factor	2.4	2.9	1.5	2.9

*: *f*, expression detected in flea but not comparison condition; *b*, expression detected in bubo but not flea; *ns*, no expression detected in flea or comparison condition.

Expression of genes in the pH 6 antigen locus (*psaEFABC*), responsible for the synthesis and transport of the PsaA fimbriae that enhance resistance to phagocytosis by macrophages [35],[36], were higher in the bubo than the flea, although the usher protein gene *psaC* was upregulated in the flea compared to *in vitro* growth (Tables 1, S1). The *psa* locus is regulated by RovA [36]. Consistent with these findings, *rovA* expression was downregulated in the flea; whereas expression of *rovM*, a negative regulator of *rovA* [37], was upregulated.

The transcriptional regulator gene *phoP* of the PhoPQ two-component regulatory system and the PhoP-regulated *mgtC* gene were expressed at levels >2-fold higher in fleas than in any other condition (Tables 1, S1, S3). PhoP and MgtC are established virulence factors known to be important for survival of *Y. pestis* and other gram-negative bacteria in macrophages and for resistance to cationic antimicrobial peptides (CAMPs) of the mammalian innate immune response [38],[39],[40]. The PhoPQ system is induced in low Mg^2+^ or low pH environments, or by exposure to CAMPs [41],[42],[43]. The Mg^2+^ concentration and pH of the flea digestive tract have not been defined, so the inducing stimulus is unknown, but CAMPs are induced and secreted into the gut by blood feeding insects when they take a blood meal containing bacteria [44],[45]. *X. cheopis* fleas encode homologs of the insect CAMPs cecropin and defensin, and mount an inducible antibacterial response to infection (unpublished data). Thus, the PhoPQ regulatory system may be induced by the flea's immune system in response to *Y. pestis* in the midgut. Despite the upregulation of *phoP* in the flea, with the notable exception of *mgtC* there was little correlation between predicted PhoP-regulated genes *in vitro* and genes upregulated in the flea [39],[46],[47]. Differential regulation of members of the PhoP regulon may occur depending on the inducing stimulus, however [48].

### Induction of a phagocytosis-resistant phenotype in the flea

**Figure 4:**
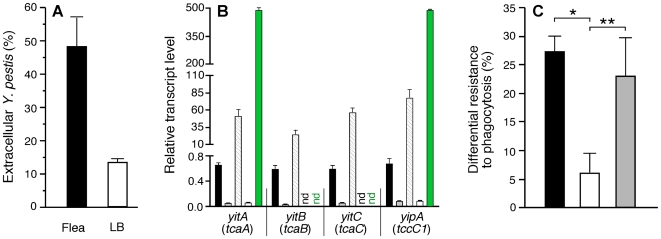
Phagocytosis-resistant phenotype of *Y. pestis* isolated from fleas correlates with expression level of the *yit*-*yip* insecticidal-like toxin genes. (A) The percentage of extracellular *Y. pestis* KIM6+ 1 hour after addition to murine bone marrow macrophages are shown for bacteria from *in vitro* cultures (LB) or from infected fleas. The mean and SEM of five independent experiments done in duplicate are shown; *P*<0.0001. (B) Relative transcript levels of insecticidal-like toxin genes in *Y. pestis* KIM6+ wt grown in LB (black bars), Δ*yitR* mutant grown in LB (grey bars), Δ*yitR* mutant from fleas (white bars), and the complemented Δ*yitR* mutant from LB (hatched bars) and from fleas (green bars); nd = not done. The mean and SEM of three independent experiments done in triplicate are shown. Values corresponding to separate segments of the y-axis are significantly different (*P*<0.001); values for LB-grown wt bacteria (black bars) are also significantly different from values represented by the grey and white bars (*P*<0.05). (C) Differential resistance to phagocytosis by murine macrophages (% extracellular flea-derived bacteria minus % extracellular *in vitro*-grown bacteria) of *Y. pestis* KIM6+ wt (black bar, n = 3), Δ*yitR* mutant (white bar, n = 3), and complemented Δ*yitR* mutant (grey bar, n = 2). The mean and standard error of the n experiments done in duplicate are indicated; *P<0.01; ** *P* = 0.06.

Soon after transmission, *Y. pestis* would be expected to encounter rapidly-responding phagocytic cells in the dermis. To assess the overall effect of the flea-specific phenotype on this encounter, we compared the interaction of *Y. pestis* recovered from infected fleas and from *in vitro* cultures with murine bone marrow macrophages. Bacteria from fleas showed significantly lower levels of phagocytosis (Fig. 4A). We have previously reported analogous findings using human polymorphonuclear leukocytes (PMNs) [7].

The *yit* and *yip* genes in a *Y. pestis* locus (y0181–0191) that encode predicted insecticidal-like toxins of the toxin complex (Tc) family and three linked phage-related genes were upregulated 4- to 50-fold in the flea midgut (Tables 1 and S1). We previously reported that the genes for these Tc-like proteins are highly expressed in fleas, but that their products are nontoxic to fleas [49]. *yitR*, a LysR-type regulator that activates the Tc-like *yit* genes [50], was upregulated >10-fold in the flea, but its expression was not detected in the rat bubo (Table 1). The specific induction in the flea of *yitR* and genes in the adjacent Tc-like *yit* and *yip* loci suggests that they are involved in adaptation to and colonization of the flea. However, deletion of *yitR* or *yitA-yipB* (y0183–y0191) does not affect the ability of *Y. pestis* KIM6+ to infect or block fleas (data not shown). These observations, and the fact that the *Yersinia* Tc proteins have toxicity to certain eukaryotic cell lines *in vitro* [50],[51], prompted us to investigate a possible post-transmission antiphagocytic role for these proteins in the mammalian host.

To determine if the insecticidal-like toxins were involved in resistance to phagocytosis, we repeated the macrophage experiments with a *Y. pestis* Δ*yitR* mutant, which as expected showed greatly reduced expression of the *yit* and *yip* genes *in vitro* and in the flea (Fig. 4B). Loss of *yitR* significantly reduced the increased resistance to phagocytosis of *Y. pestis* isolated from infected fleas (Fig. 4C).

**Figure 5:**
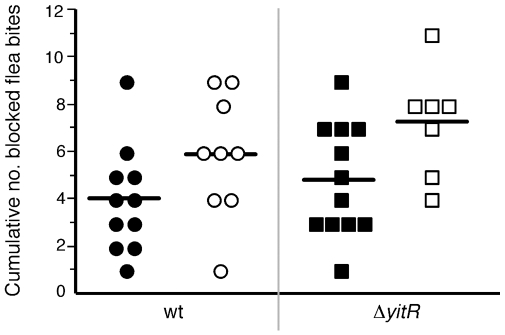
Mean and range of the cumulative number of blocked flea bites received by mice. Circles and squares indicate individual mice challenged by fleas infected with wt or Δ*yitR Y. pestis* 195/P, respectively. Filled symbols indicate mice that developed terminal plague; open symbols indicate mice that did not develop disease.

Since the *yit* and *yip* genes are not required for *Y. pestis* to produce a transmissible infection in fleas, it was possible to compare the virulence of wild-type and Δ*yitR Y. pestis* following transmission by fleabite. The incidence rate and time to disease onset were identical for both *Y. pestis* strains, demonstrating that expression of *yit* and *yip* is not essential for flea-borne transmission or disease (data not shown). On average, the mice challenged with *Y. pestis* Δ*yitR*-infected fleas, both those that developed disease and those that did not, received a higher cumulative number of bites from blocked fleas than the mice challenged with *Y. pestis*-infected fleas, but this difference was not statistically significant (Fig. 5). However, it was not possible to detect any relatively minor difference in LD50 because the number of bacteria transmitted by a blocked flea varies widely [1],[52]. Even a small decrease in LD50 provided by the Yit-Yip proteins would be significant at the ecological level in the maintenance of plague transmission cycles, because the transmission efficiency of blocked fleas is very low– often only a few or no bacterial cells are transmitted in an individual fleabite [52]. Because *phoP* is required by *Y. pestis* to produce a transmissible infection in fleas (unpublished data), it was not possible to similarly assess the effect on disease transmission of *phoP* induction in the flea.

### Does transit through the flea vector preadapt *Y. pestis* to resist mammalian innate immunity?

When *Y. pestis* is transmitted into the dermis by an infected flea, it is immediately exposed to the mammalian innate immune system. The most important antiphagocytic virulence factors, the cytotoxic *Yersinia* outer proteins (Yops), part of the T3SS encoded by the *Y. pestis* virulence plasmid and the F1 capsule encoded by the pMT1 plasmid, are not present at this initial stage of infection. Their expression is strictly temperature-regulated and are not produced *in vivo* until 3–5 hours after the temperature shift to 37°C that accompanies transmission [1],[3],[53],[54]. Consequently, *Y. pestis* grown at <28°C *in vitro* are initially susceptible to in *vivo* uptake and killing by phagocytes until the Yop and F1 virulence factors are produced, effectively preventing further phagocytosis [53],[54]. Our results indicate that *Y. pestis* entering the mammal from an infective flea is relatively resistant to macrophages, as well as PMNs [7]; a vector-specific phenotype that is not related to the T3SS or capsule.

Coming from the flea, *Y. pestis* is also associated with the biofilm ECM, identical or closely related to the poly-β-1,6-*N*-acetyl glucosamine ECM of staphylococcal biofilms, which has been shown to provide protection from innate immune components [55],[56]. In addition, although the antiphagocytic F1 capsule and Psa fimbriae do not appear to be produced in the flea, upregulation in the flea of most F1 genes in the *cafRcaf1M1A1* locus and the Psa usher protein gene *psaC* (Tables 1, S1) suggests that components of the F1 and Psa translocation system are made, which may prime *Y. pestis* for rapid secretion of these extracellular virulence factors after transmission. The upregulation of the innate immunity resistance genes *phoP* and *mgtC* suggest that those *Y. pestis* that are phagocytized may be prepared for resistance to CAMPs and intracellular survival while still in the flea vector. Finally, the major essential virulence factors *yadBC* and *pla*, essential for *Y. pestis* dissemination from the dermis, were maximally or very highly expressed in the flea (Tables 1, S3). Besides degrading plasminogen, the Pla protease may also inactivate CAMPs, particularly when the F1 capsule is not present [57], which matches the phenotype of *Y. pestis* in the flea.

In summary, *Y. pestis* appears to be prepared for pathogenesis in the mammal while still in the flea vector. The biofilm phenotype of *Y. pestis* and the virulence factors upregulated or highly expressed in the flea may enhance the earliest stages of plague pathogenesis while the full complement of temperature-shift-regulated virulence factors is still being induced. Increased resistance to innate immunity that is preinduced in the flea vector may be critical to productive transmission because blocked fleas transmit relatively few bacteria, often below the LD50 of *Y. pestis* grown *in vitro* at <28°C [1],[52].

## Materials and Methods

### Ethics statement

All animals were handled in strict accordance with good animal practice as defined by NIH animal care and use policies and the Animal Welfare Act, USPHS; and all animal work was approved by the Rocky Mountain Laboratories Animal Care and Use Committee.

### Bacterial strains and growth conditions for *in vitro* transcriptome analyses

*Y. pestis* KIM6+, which lacks the 70-kb virulence plasmid that is not required for flea infection or blockage, was used for gene expression analyses. A KIM6+ strain with an in-frame deletion that eliminated amino acids 28–281 of the predicted 291 amino acid residue *yitR* (y0181) gene product was produced by allelic exchange, using the pCVD442 suicide vector system [11]. This mutant was complemented by electroporation with a recombinant pWKS130 plasmid containing the wild-type *yitR* promoter and orf. The Δ*yitR* mutant was also transformed with pWKS130 alone to generate an empty vector control strain. For *in vitro* planktonic samples, bacteria were grown from frozen stocks in brain heart infusion (BHI) medium at 28°C, followed by two successive transfers in Luria Bertani broth supplemented with 100 mM MOPS, pH 7.4 (LB/MOPS) at 21°C. An inoculum of 10^4^ cells/ml was added to 50 ml of LB/MOPS and incubated at 21°C with shaking at 250 rpm until exponential (OD_600_ = 2.5) or stationary phase (OD_600_ = 4.5). Approximately 0.5 ml of the exponential phase culture and 0.25 ml of the stationary phase culture was resuspended in 1 ml and 0.5 ml, respectively, of RNAprotect bacterial reagent (Qiagen; Valencia, CA), incubated for 5 min at room temperature, and centrifuged at 21°C for 5 min prior to RNA extraction.

For *in vitro* biofilms, 400 µl of a 10^7^/ml bacterial suspension was injected into a flowcell (Stovall; Greensboro, NC) that was connected to a reservoir of LB/MOPS at 21°C. Following a 30 min incubation period to allow the bacteria to adhere to the glass surface of the flow cell, LB/MOPS was pumped through the flow cell at a rate of 0.3 ml/min. After 48 hours, the flowcell was disconnected and the thick *Y. pestis* biofilm was harvested and treated with 0.5ml of RNAprotect similarly to the planktonic cultures.

### Flea infections and collection of samples for *in vivo* transcriptome analyses

*X. cheopis* fleas were infected with *Y. pestis* KIM6+ by using a previously described artificial feeding system [3]. The infectious blood meal was prepared by growing *Y. pestis* KIM6+ overnight at 37°C in BHI medium, without aeration. A cell pellet containing 10^9^ bacterial cells was resuspended in 1 ml PBS and added to 5 ml heparinized mouse blood. Fleas that took a blood meal were maintained at 21°C and 75% relative humidity, fed twice weekly on uninfected mice, and monitored for proventricular blockage as previously described [3]. On the day blockage was diagnosed, the digestive tract was dissected out and macerated in RNAprotect, a process that required about 1 min. Thirty midguts from blocked fleas were pooled for each of the two biological replicates. Midguts from 60 uninfected fleas were also collected as controls to assess background hybridization of flea RNA to the microarray.

A flea-borne transmission model [58] was used to determine *Y. pestis* infectivity after challenge by flea bite. Fleas were infected with *Y. pestis* 195/P, a fully virulent wild-type strain, or with a *Y. pestis* 195/P Δ*yitR* mutant constructed as described above. Between 2–3 weeks after infection, the time required for *Y. pestis* to block fleas with a proventricular biofilm, groups of 20–40 fleas were applied to a restrained mouse and allowed to feed for 60 min. The fleas were then recovered and examined under a dissecting microscope to determine how many had taken a normal blood meal (unblocked or non-infective fleas) and how many were blocked (infective fleas). After challenge, mice were monitored and euthanized upon the appearance of signs of terminal illness. Mice that did not develop any symptoms after one week following a challenge were re-challenged. A total of 9–10 BALB/cAnN and 10 RML Swiss-Webster mice were challenged with each strain.

### RNA isolation, amplification, and microarray

RNA was isolated from six independent samples from *in vitro* and flow cell cultures and two independent samples from pooled blocked fleas (Fig. S1) using the RNeasy Mini Kit (Qiagen). Flea-derived RNA samples were secondarily split into three technical replicates each. RNA integrity was verified on a Bioanalyzer 2100 (Agilent Technologies; Santa Clara, CA). Total RNA (100 ng) was amplified and labeled with modified biotin-11-CTP (Perkin Elmer; Waltham, MA) and biotin-16-UTP (Roche Molecular Biochemicals, Pleasanton, CA) by using the Message-Amp II-Bacteria amplified antisense RNA (aRNA) kit (Ambion; Austin, TX). Amplified RNA was then fragmented using Ambion's Fragmentation reagent (Applied Biosystems), hybridized to the RML custom Affymetrix GeneChip that contains sequences for all *Y. pestis* predicted ORFs, and scanned. The amplification step did not affect the relative transcript signals obtained by microarray (data not shown).

### Microarray data analysis

Affymetrix GeneChip Operating Software (GCOS v1.4, GEO platform GPL2129, http://www.affymetrix.com) was used for initial analysis of the microarray data at the probe-set level. All *.*cel* files, representing individual biological replicates, were scaled to a trimmed mean of 500 using a scale mask consisting of only the *Yersinia pestis* KIM6+ probe-sets to produce the *.*chp* files. A pivot table with all samples was created including calls, call p-value and signal intensities for each gene. The pivot table was then imported into GeneSpring GX 7.3 (http://www.chem.agilent.com), where hierarchical clustering (condition tree) using a Pearson correlation similarity measure with average linkage was used to produce the dendrogram indicating that biological replicates grouped together. The pivot table was also imported into Partek Genomics Suite software (Partek Inc.; St. Louis, MO) to produce a principal components analysis (PCA) plot as a second statistical test for the grouping of biological replicates. ANOVA was run from this data set to produce a false discovery rate report producing false positive reduced p-values for each comparison of interest.

The correlated replicates of all test conditions and controls were combined, and quality filters based upon combined calls and signal intensities were used to further evaluate individual gene comparisons. Present and marginal calls were treated as the same whereas absent calls were negatively weighted and eliminated from calculations. Ratios of test/control values and associated t-test and ANOVA p-values values of all individual genes passing the above filters were determined using GeneSpring, SAM, and Partek software. The microarray data have been deposited in the NCBI GEO public database (accession number GSE16493).

To compare differential *in vivo* gene expression patterns in the flea and the rat, the average hybridization signal for each individual *Y. pestis* gene was divided by the average signal of all 4,683 genes on the microarray for both the flea microarray (this study) and the rat bubo microarray [11] data sets. Gene by gene comparisons of these normalized expression data sets were used for Fig. 3 and Tables 1, S5, and S6).

### Macrophage phagocytosis assay

Murine bone marrow-derived macrophages were prepared as described [59],[60] and cultured in Dulbecco's Modified Eagles medium (DMEM) supplemented with 5 mM L-glutamine, 25 mM HEPES, 10% heat-inactivated fetal bovine serum, 5 mM non-essential amino acids, and 10 ng/ml CSF-1 (PeproTech; Rocky Hills, NJ). 1-ml suspensions of *Y. pestis* KIM6+ containing pAcGFP1 (Clontech; Mountain View, CA) from 21°C stationary phase LB/MOPS cultures, or from triturated midguts dissected from fleas 2 to 3 weeks after infection were treated for 15 sec in a FastPrep FP120 using lysing matrix H (Qbiogene; Carlsbad, CA) to disrupt bacterial aggregates, quantified by Petroff-Hausser direct count, and diluted in DMEM to ∼1×10^6^ bacteria/ml. 0.1 ml of bacterial suspension was added to tissue culture plate wells containing ∼1×10^5^ differentiated primary macrophages cultured on 12 mm glass coverslips in 1 ml DMEM. The plates were not centrifuged after addition of the bacteria, and midgut triturate from an equivalent number of uninfected fleas was added to the *in vitro*-derived bacterial suspensions used for these experiments. After 1 h incubation at 37°C and 5% CO_2_, the medium was removed and the cells washed, fixed in 2.5% paraformaldehyde for 10 min at 37°C, and then rewashed. Extracellular bacteria were labelled by indirect immunofluorescence as described [60] using a 1∶50,000 dilution of hyperimmune rabbit anti-*Y. pestis* polyclonal antibody [7] and a 1∶400 dilution of AlexaFluor 568-conjugated goat anti-rabbit antibody (Invitrogen; Carlsbad, CA). The percentage of extracellular bacteria was determined by dividing the number of red-fluorescent bacteria by the total number (red- and green only-fluorescent) bacteria associated with individual macrophages. To calculate differential resistance to phagocytosis for a given strain, the average percent extracellular LB-grown bacteria was subtracted from the average percent extracellular flea-derived bacteria. Results from 2–3 independent experiments performed in triplicate were analyzed by unpaired two-tailed t-test.

### Quantitative RT-PCR

Independent RNA samples were prepared from blocked fleas and *in vitro* biofilm and planktonic cultures as described for the microarray experiments, except that the RNA was not amplified. Samples were treated with rDnase I (Ambion) and confirmed by PCR to be free of genomic DNA contamination. cDNA was synthesized from the RNA and used for quantitative PCR on an ABI Prism 7900 sequence detection system (Taqman, Applied Biosystems). The reactions contained oligonucleotide primers and probes designed using Primer Express version 2.0 software (Applied Biosystems) and the Taqman Universal PCR Master Mix (Applied Biosystems). For each primer-probe set assay, a standard curve was prepared using known concentrations of *Y. pestis* KIM6+ genomic DNA and used to transform C_T_ values into relative DNA quantity. The quantity of cDNA for each experimental gene was normalized relative to the quantity of the reference gene *crr* (y1485), and the ratio of the normalized quantity of each gene in the flea samples to the normalized quantity in the *in vitro* samples was calculated (Fig. S2). Primer and probe sets used are listed in Table S7.

## Supporting Information

Figure S1 Representative electrophoretograms of total RNA extracted from dissected flea digestive tracts. Electrophoretograms derived from uninfected (A) and blocked (B) flea digestive tracts are shown, with prokaryotic and eukaryotic rRNA peaks indicated.
(0.97 MB TIF)

Figure S2 Quantitative reverse transcription (QRT) PCR confirmation of microarray results. The quantity of each mRNA was determined relative to that of the reference gene *crr* (y1485). Fold-differences in transcript levels of the 12 *Y. pestis* genes in the flea compared to (A) *in vitro* biofilm, (B) exponential phase planktonic cultures, and (C) stationary phase planktonic cultures are shown as determined by microarray (grey bars) and QRT-PCR (black bars). *gabT transcript was detected by microarray in the flea samples only.
(1.92 MB TIF)

Table S1 *Y. pestis* genes upregulated ≥2-fold in the flea relative to all *in vitro* conditions.
(0.32 MB DOC)

Table S2 *Y. pestis* genes downregulated ≥2-fold in the flea relative to all *in vitro* conditions.
(0.14 MB DOC)

Table S3 The 100 most highly expressed *Y. pestis* genes in the flea.
(0.22 MB DOC)

Table S4 *Y. pestis* genes upregulated ≥2-fold in the flea and flowcell biofilms relative to planktonic culture conditions.
(0.12 MB DOC)

Table S5 *Y. pestis* genes with significantly higher relative expression levels in the flea gut than in the rat bubo.
(0.28 MB DOC)

Table S6 *Y. pestis* genes with significantly higher relative expression levels in the rat bubo than in the flea.
(0.51 MB DOC)

Table S7 Primers and probes used for quantitative RT-PCR.
(0.06 MB DOC)
