# Supplementary figures and images for: Transit through the Flea Vector Induces a Pretransmission Innate Immunity Resistance Phenotype in Yersinia pestis
Source: PLoS Pathog. 2010 Feb 26;6(2):e1000783. doi: 10.1371/journal.ppat.1000783 (PMC2829055; doi:10.1371/journal.ppat.1000783)

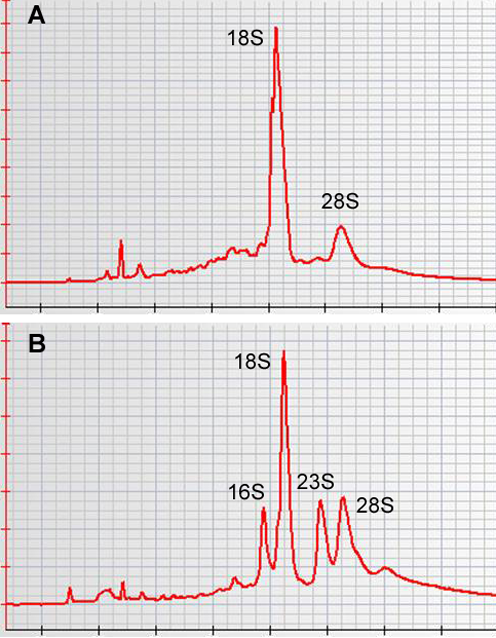

Supplement: Figure S1 — Representative electrophoretograms of total RNA extracted from dissected flea digestive tracts. Electrophoretograms derived from uninfected (A) and blocked (B) flea digestive tracts are shown, with prokaryotic and eukaryotic rRNA peaks indicated. (0.97 MB TIF) [file ppat.1000783.s001.tif]

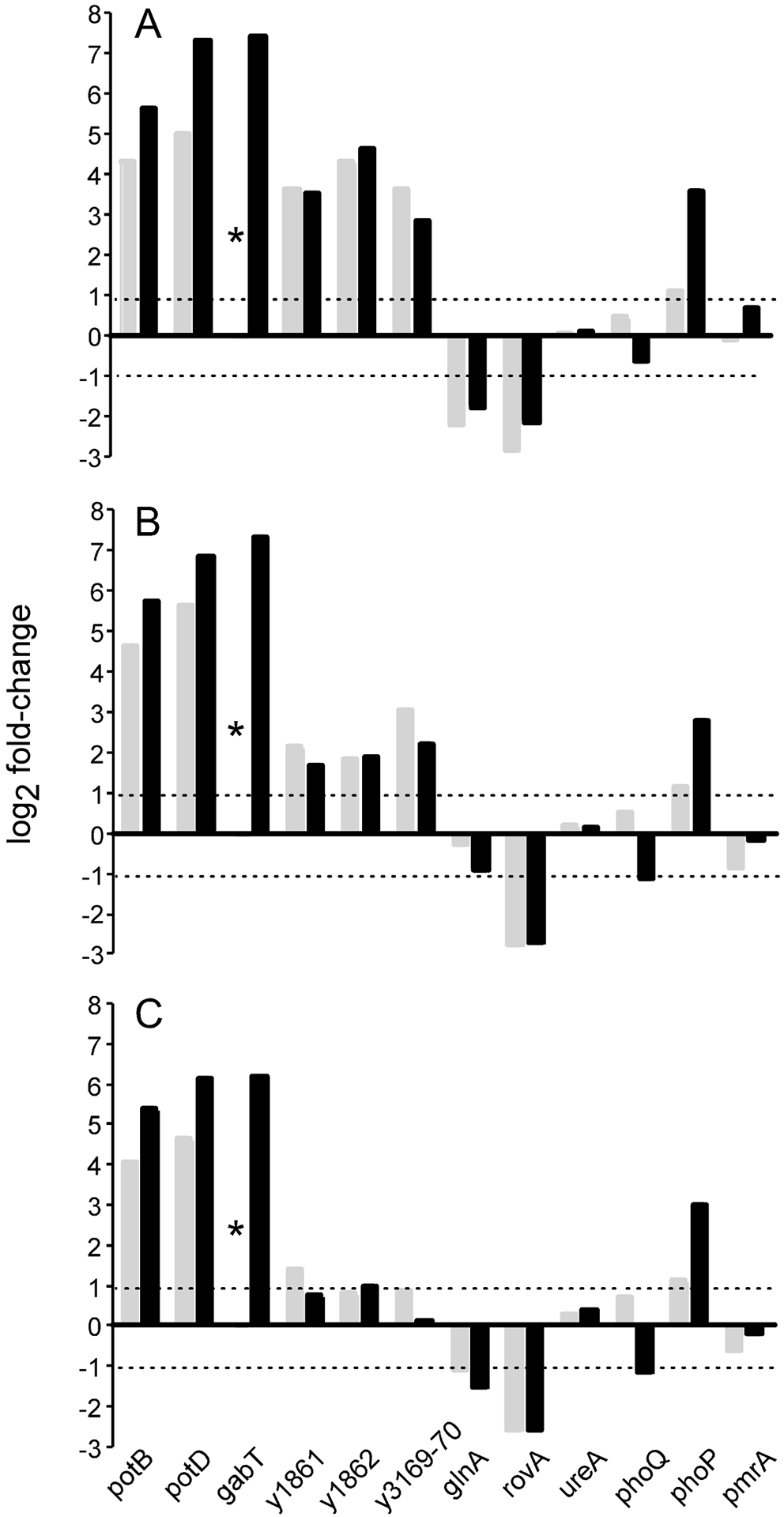

Supplement: Figure S2 — Quantitative reverse transcription (QRT) PCR confirmation of microarray results. The quantity of each mRNA was determined relative to that of the reference gene crr (y1485). Fold-differences in transcript levels of the 12 Y. pestis genes in the flea compared to (A) in vitro biofilm, (B) exponential phase planktonic cultures, and (C) stationary phase planktonic cultures are shown as determined by microarray (grey bars) and QRT-PCR (black bars). *gabT transcript was detected by microarray in the flea samples only. (1.92 MB TIF) [file ppat.1000783.s002.tif]
