# Supplementary material for: Transit through the Flea Vector Induces a Pretransmission Innate Immunity Resistance Phenotype in Yersinia pestis
Source: PLoS Pathog. 2010 Feb 26;6(2):e1000783. doi: 10.1371/journal.ppat.1000783 (PMC2829055; doi:10.1371/journal.ppat.1000783)
Supplement: Table S1 — Y. pestis genes upregulated ≥2-fold in the flea relative to all in vitro conditions. (0.32 MB DOC) [file ppat.1000783.s003.doc]

**Table S1.** *Y. pestis* genes upregulated ≥ 2-fold in the flea relative to all *in vitro* conditions

| **gene** | **orf** | **predicted function of gene product** | **fold change* relative to:** | | | | |
| --- | --- | --- | --- | --- | --- | --- | --- |
|  |  |  | **flowcell biofilm** | **exp. phase planktonic** | | **stat. phase planktonic** | |
| **A. Plasmid genes** | | | | | | | |
| - | Y1043 | hypothetical protein | 2.8 | | 3.0 | | 2.7 |
| *-* | Y1067 | hypothetical protein | 4.5 | | 5.4 | | 5.0 |
| *parA* | Y1076.S | partitioning protein | 3.2 | | 3.3 | | 3.3 |
| *parB* | Y1077 | partitioning protein | 2.9 | | 2.4 | | 2.3 |
| *caf1R* | Y1097 | F1 operon positive regulatory protein | *f* | | *f* | | *f* |
| *caf1M* | Y1098 | F1 chaperone protein | *f* | | *f* | | *f* |
| *caf1A* | Y1099 | F1 capsule anchoring protein | 7.1 | | 5.3 | | 4.0 |
| *caf1* | Y1100 | F1 capsule antigen | 6.8 | | 5.0 | | 4.5 |
|  | | | | | | | |
| **B. Chromosomal genes** | | | | | | | |
| *Amino acid transport and metabolism* | | | | | | | |
| - | y0932 | solute binding protein of amino acid ABC transporter | *f* | | 14.3 | | 16.7 |
| *-* | y0933 | putative amino acid ABC transporter permease | *f* | | *f* | | *f* |
| *-* | y0934 | permease of ABC transporter | *f* | | 7.1 | | 5.6 |
| *-* | y1147 | malonic semialdehyde oxidative decarboxylase | 4.9 | | 2.0 | | 2.7 |
| *gabT* | y1390 | γ aminobutyrate aminotransferase | *f* | | *f* | | *f* |
| *potD* | y1391 | spermidine-preferential substrate-binding protein of ABC transporter | 33.8 | | 43.1 | | 25.8 |
| *potB* | y1392 | spermidine- preferential permease of ABC transporter | 21.0 | | 24.4 | | 17.6 |
| *potC* | y1393 | putative ABC transporter permease | *f* | | 11.1 | | 10.0 |
| *hisQ* | y1608 | histidine ABC transport system inner membrane permease | 5.4 | | 2.5 | | 4.7 |
| *glnQ* | y1673 | glutamine ABC transporter ATP-binding component | 8.9 | | 6.2 | | 9.1 |
| *glnP* | y1674 | glutamine ABC transporter permease component | 15.4 | | 4.4 | | 9.6 |
| *glnH* | y1675 | glutamine ABC transporter periplasmic-binding protein | 13.8 | | 4.7 | | 9.3 |
| *-* | y2275 | putative histidinol phosphatase hypothetical protein | 3.6 | | 3.1 | | 2.1 |
| *hutI* | y2340 | imidazolonepropionase | 13.0 | | 5.3 | | 3.7 |
| *hutG* | y2341 | histidine degradation enzyme | 10.8 | | 4.5 | | 2.9 |
| *putA* | y2455 | proline dehydrogenase, P5C dehydrogenase | 3.9 | | 5.5 | | 4.8 |
| *hpaC* | y2538 | hypothetical protein | 5.1 | | 4.7 | | 2.5 |
| *hpaB* | y2539 | component B of 4-hydroxyphenylacetic acid-hydroxylase | 6.7 | | 4.5 | | 2.5 |
| *hpaX* | y2540 | 4-hydroxyphenylacetate permease | 6.5 | | 4.8 | | 3.4 |
| *hpaI* | y2541 | 2,4-dihydroxyhept-2-ene-1,7-dioic acid aldolase | *f* | | 5.5 | | 3.7 |
| *hpaH* | y2542 | 2-oxo-hept-3-ene-1,7-dioate hydratase | 9.0 | | 4.7 | | 3.5 |
| *hpaF* | y2544 | 5-carboxymethyl-2-hydroxymuconate isomerase | 11.5 | | 5.9 | | 4.4 |
| *hpaD* | y2545 | 3,4-dihydroxyphenylacetate 2,3-dioxygenase | *f* | | 5.9 | | 3.8 |
| *hpaE* | y2546 | 5-carboxymethyl-2-hydroxymuconate semialdehyde dehydrogenase | *f* | | 3.4 | | 3.7 |
| *ansB* | y2787 | periplasmic L-asparaginase II | 16.7 | | 24.7 | | 18.0 |
| *artQ* | y2831 | arginine 3rd transport system permease | 2.7 | | 6.2 | | 3.4 |
| *gabD* | y2894 | succinate-semialdehyde dehydrogenase | 20.3 | | 5.8 | | 6.9 |
| - | y2896 | putative *D*-2-hydroxyacid dehydrogenase family protein | 4.6 | | 4.7 | | 3.8 |
| *glnE* | y3525 | adenylylating enzyme for glutamine synthetase | 2.3 | | 2.2 | | 2.1 |
| *proY* | y4036 | aromatic amino acid permease | 2.3 | | 3.2 | | *f* |
| *hutH* | y4037 | histidine ammonia-lyase | 20.0 | | 5.3 | | 3.5 |
| *hutU* | y4038 | urocanate hydratase | 8.5 | | 6.0 | | 4.0 |
|  | | | | | | | |
| *Carbohydrate transport and metabolism* | | | | | | | |
| *-* | y0329 | putative sugar transport system ATP-binding component | 3.9 | | 2.5 | | 2.4 |
| *-* | y0857 | Ribose/xylose/arabinose/galactoside ABC-type | 4.3 | | 3.0 | | 2.9 |
| *rbsA* | y1150 | ATP-binding component of D-ribose high-affinity transport system | 3.9 | | 2.4 | | 2.4 |
| *rbsC* | y1151 | D-ribose high-affinity transport system | 4.4 | | 4.6 | | 4.1 |
| *iolB* | y1153 | myo-inositol catabolism protein | *f* | | 3.4 | | 3.8 |
| *chbB* | y1251 | chitobiose PTS system transporter subunit IIB | 4.4 | | 3.2 | | 6.1 |
| *chbC* | y1252 | chitobiose PTS system transporter subunit IIC | 4.4 | | 2.7 | | 4.4 |
| *araH* | y2098 | high-affinity L-arabinose transport system membrane protein | 3.2 | | 4.4 | | 2.8 |
| *-* | y2272 | permease | 2.3 | | 3.7 | | 2.9 |
| *dctQ* | y2586 | putative C4-dicarboxylate transporter, small subunit | 3.1 | | 2.1 | | 2.5 |
| *dctM1* | y2587 | putative C4-dicarboxylate transporter, large subunit | 6.0 | | 5.5 | | 5.7 |
|  | y2591 | polysaccharide deacetylase | 2.8 | | 2.8 | | 2.2 |
| - | y2592 | sugar transporter | *f* | | 4.5 | | 3.4 |
| *-* | y2602 | transport protein | 7.6 | | 3.8 | | 4.4 |
| *mglA* | y2661 | ABC transporter and galactose taxis | 4.1 | | 2.0 | | 5.4 |
| *-* | y2889 | ribose ABC transporter permease | *f* | | 4.0 | | 2.9 |
| *rpiA* | y2892 | ribose 5-phosphate isomerase A | 4.9 | | 2.9 | | 3.0 |
| *-* | y2893 | xylulose kinase | *f* | | 5.9 | | 6.7 |
| *-* | y2961 | oligogalacturonide transporter | 5.2 | | 5.6 | | 5.8 |
| *-* | y3222 | PTS permease protein | 2.7 | | 3.5 | | 4.9 |
| *ugpB* | y3566 | solute-binding periplasmic protein of ABC transporter | 3.2 | | 2.7 | | 2.1 |
| *-* | y3569 | ATP-binding component of sn-glycerol 3-phosphate transport system | 3.3 | | 2.7 | | 2.8 |
| *ptsA* | y3776 | PEP-protein phosphotransferase system enzyme I | 6.1 | | 4.7 | | 3.6 |
| *frwC* | y3777 | PTS system fructose-like 2IIC component | 9.2 | | 11.1 | | 8.9 |
| *frwB* | y3779 | PTS system fructose-like IIB component | *f* | | 11.1 | | 10.0 |
| *frwD* | y3780 | PTS system fructose-like IIB component | 8.6 | | 7.4 | | 9.9 |
| *chbF* | y3950 | phospho-β-glucosidase | 4.6 | | 4.0 | | 2.8 |
| *mdfA* | y4067 | proton motive force efflux pump protein | 2.9 | | 4.4 | | 2.5 |
|  | | | | | | | |
| *Cell motility* | | | | | | | |
| *motA* | y1823 | flagellar motor protein | 3.6 | | 4.1 | | 2.4 |
| *cheA* | y1827 | sensory transducer kinase | 4.8 | | 5.2 | | 3.5 |
| *fliZ* | y2463 | hypothetical protein | 2.2 | | 3.5 | | 2.6 |
| *fliT* | y2468 | repressor of class 3a and 3b operons (RflA activity) | 3.0 | | 3.2 | | 3.7 |
| *fliP* | y2486 | flagellar biosynthesis protein | 3.2 | | 4.5 | | 2.6 |
| *tsr2* | y2958 | methyl-accepting chemotaxis transmembrane protein | 2.3 | | 2.4 | | 2.1 |
|  | | | | | | | |
| *Cell wall/membrane biogenesis* | | | | | | | |
| *lpxP* | y0236 | lipid A lauroyl acyltransferase | 2.2 | | 3.4 | | 3.2 |
| *yapL* | y1454 | putative autotransporter, similar to *tibA* | 2.2 | | 3.3 | | 2.0 |
| - | y1806 | oxidoreductase | 5.0 | | 3.0 | | 2.6 |
| *alr* | y1849 | alanine racemase | 3.2 | | 3.8 | | 3.5 |
| *mltE* | y1898 | murein transglycosylase E | 2.3 | | 2.1 | | 2.4 |
| *ompW* | y2044 | outer membrane protein | 6.4 | | 24.9 | | 21.9 |
| *-* | y2333 | nucleotide di-P-sugar epimerase or dehydratase | 2.9 | | 3.6 | | 2.1 |
| - | y2598 | putative racemase | 5.2 | | 2.1 | | 2.0 |
|  | | | | | | | |
| *Defense mechanisms* | | | | | | | |
| - | y2365 | integral membrane protein | 3.9 | | 3.7 | | 2.9 |
|  | | | | | | | |
| *Energy production and conversion* | | | | | | | |
| - | y1651 | putative *D*-2-hydroxyacid dehydrogenase family protein | 2.4 | | 2.7 | | 2.0 |
| - | y2085 | electron transport complex protein RnfC | 2.1 | | 3.3 | | 2.5 |
| *hcp* | y2819 | hydroxylamine reductase | 2.7 | | 6.1 | | 3.3 |
|  | | | | | | | |
| *Extracellular structures* | | | | | | | |
| - | y1846 | surface protein | 4.4 | | 5.4 | | 2.0 |
|  | | | | | | | |
| *Inorganic ion transport and metabolism* | | | | | | | |
| *hmuU* | y0540 | hemin ABC-transporter permease | 2.0 | | 3.0 | | 2.3 |
| *yfuB* | y1525 | inner membrane permease of iron ABC transporter | 3.2 | | 3.1 | | 2.8 |
| *yfuA* | y1526 | solute-binding periplasmic protein for iron ABC transporter | 4.5 | | 4.4 | | 2.4 |
| *-* | y2112 | putative chloride channel | 2.8 | | 5.5 | | 4.1 |
| *-* | y2370 | integral membrane protein | 3.0 | | 4.9 | | 4.6 |
| *yscU* | y2636 | inner membrane permease of iron ABC transporter | *f* | | *f* | | 3.1 |
| *modB* | y3036 | molybdate transport permease protein | 2.2 | | 2.2 | | 3.3 |
| *-* | y3435 | hypothetical protein | 5.8 | | 5.5 | | 2.9 |
| - | y4045 | ferric anguibactin transport system permease | 2.8 | | 3.3 | | 3.0 |
|  | | | | | | | |
| *Intracellular trafficking and secretion* | | | | | | | |
| *tatE* | y1170 | twin arginate translocase protein E | 2.8 | | 3.9 | | 2.4 |
|  | | | | | | | |
| *Adhesins and surface antigens* | | | | | | | |
| *fimD* | y0350 | fimbrial usher, *cupA3* homolog | 3.4 | | 2.7 | | 2.0 |
| - | y0352 | fimbrial subunit, *cupA1* homolog | 2.9 | | 4.0 | | 2.9 |
| - | y1860 | fimbrial subunit protein | *f* | | 5.0 | | 2.2 |
| - | y1861 | fimbrial subunit protein | 13.1 | | 4.5 | | 2.6 |
| *psaC* | y2880 | pH 6 antigen fimbrial usher | 3.4 | | 3.4 | | 2.5 |
| - | y4063 | fimbrial protein | *f* | | 6.2 | | 2.7 |
|  | | | | | | | |
|  | | | | | | | |
| *Lipid transport and metabolism* | | | | | | | |
| - | y1117 | putative acyltransferase | 2.1 | | 5.0 | | 5.7 |
| *lipB* | y1173 | lipoyltransferase | 3.7 | | 5.0 | | 3.3 |
| - | y2334 | 3-oxoacyl-(acyl-carrier-protein) synthase III | 2.5 | | 3.3 | | 2.1 |
| - | y3641 | fatty acid biosynthesis protein | 3.2 | | 3.2 | | 2.6 |
|  | | | | | | | |
| *Insertion elements* | | | | | | | |
| - | y4050 | transposase | 2.7 | | 2.8 | | 3.5 |
|  | | | | | | | |
| *Posttranslational modification, protein turnover, chaperones* | | | | | | | |
| - | y0535 | Predicted redox protein, regulator of disulfide bond formation | 4.7 | | 3.1 | | 3.7 |
| *ibpB* | y4101 | heat shock protein | 2.9 | | 2.2 | | 2.6 |
|  | | | | | | | |
| *Replication, recombination and repair* | | | | | | | |
| *mutM* | y0090 | formamidopyrimidine-DNA glycosylase | 3.2 | | 2.4 | | 2.1 |
|  | | | | | | | |
| *Secondary metabolites biosynthesis, transport and catabolism* | | | | | | | |
| - | y2599 | 2-hydroxyhepta-2,4-diene-1,7-dioate isomerase | 14.7 | | 4.4 | | 6.0 |
| *ucpA* | y2600 | oxidoreductase | 20.5 | | 8.9 | | 9.4 |
|  | | | | | | | |
| *Signal transduction mechanisms* | | | | | | | |
| *cpxA* | y0069 | hypothetical protein | 3.7 | | 5.8 | | 4.1 |
| *phoP* | y1794 | transcriptional regulatory protein | 2.1 | | 2.2 | | 2.2 |
|  | | | | | | | |
| *Transcription* | | | | | | | |
| *slmA* | y0095 | nucleoid occlusion protein | 2.4 | | 2.1 | | 2.1 |
| *yitR* | y0181 | transcriptional regulator | 10.3 | | 47.2 | | 23.4 |
| - | y0260 | transcriptional repressor in putative T6SS (IAHP) locus | 3.0 | | 2.5 | | 2.8 |
| *sfsB* | y1140 | regulator for maltose metabolism | 4.5 | | 4.3 | | 3.6 |
| *rovM* | y1629 | NADH dehydrogenase transcriptional regulator, LysR family | 9.2 | | 7.2 | | 5.9 |
| *-* | y1959 | TetR-family transcriptional regulator | 4.8 | | 3.1 | | 3.8 |
| *pspA* | y1980 | phage shock protein A | 3.9 | | 3.5 | | 2.6 |
| *-* | y2350 | multi modular; transcriptional regulator; also ATP-binding component of a transport system | 2.0 | | 2.1 | | 2.0 |
| *-* | y2950 | DEOR-type transcriptional regulator | 2.2 | | 2.3 | | 2.8 |
| *gcvA* | y3154 | positive regulator of *gcv* operon | 2.5 | | 3.2 | | 2.6 |
| *galR* | y3183 | repressor of *galETK* operon | 2.1 | | 2.3 | | 2.7 |
| *-* | y3249 | PadR-like transcriptional regulator | 2.5 | | 4.1 | | 4.1 |
|  | | | | | | | |
| *General function prediction and function unknown* | | | | | | | |
| - | y0177 | hypothetical protein | 2.6 | | 2.1 | | 3.5 |
| *-* | y0440 | efflux pump | 2.2 | | 2.0 | | 2.6 |
| *actP* | y0508 | acetate permease | 26.2 | | 2.1 | | 4.1 |
| *-* | y0509 | putative inner membrane protein | *f* | | 3.0 | | 4.8 |
| *acs* | y0510 | acetyl coenzyme A synthetase | *f* | | 4.0 | | 3.8 |
| *-* | y0534 | integral membrane protein | 3.1 | | 2.6 | | 3.6 |
| - | y0700 | putative monooxygenase | 4.0 | | 3.5 | | 2.7 |
| *yddG* | y0773 | drug efflux pump | 2.4 | | 2.4 | | 2.7 |
| - | y0949 | hypothetical protein | 6.8 | | 4.2 | | 2.3 |
| *cof* | y1036 | haloacid dehalogenase-like hydrolase | 3.5 | | 2.8 | | 3.2 |
| - | y1288 | enzyme | 2.9 | | 2.6 | | 2.3 |
| - | y1330 | hypothetical protein | 2.1 | | 3.3 | | 2.3 |
| - | y1474 | hypothetical protein | 2.2 | | 3.6 | | 2.9 |
| - | y1555 | VgrG-like PT-rich protein in putative T6SS (IAHP) locus | 4.7 | | 3.0 | | 2.0 |
| *-* | y1595 | hypothetical efflux pump | 3.3 | | 3.8 | | 2.6 |
| - | y1698 | hypothetical protein | 4.3 | | 8.3 | | 4.1 |
| *mgtC* | y1820 | modulator of P-type ATPase | 3.0 | | 5.7 | | 4.4 |
| - | y1835 | hypothetical protein | 2.1 | | 2.9 | | 3.2 |
| - | y2367 | integral membrane protein | 3.0 | | 2.8 | | 2.5 |
| *-* | y2857 | TrkA, Potassium channel-family protein | 2.0 | | 2.0 | | 2.1 |
| *-* | y2878 | predicted inner membrane protein *yeiH* | 10.8 | | 11.6 | | 9.2 |
| *-* | y2999 | hypothetical ABC transporter ATP-binding protein | 2.7 | | 3.3 | | 3.0 |
| - | y3228 | hypothetical protein | 2.4 | | 2.5 | | 2.7 |
| *mdaB* | y3509 | modulator of drug activity B | 2.2 | | 2.2 | | 3.9 |
| - | y3519 | hypothetical protein | 2.4 | | 3.4 | | 3.0 |
| - | y3654 | hypothetical protein | 2.2 | | 2.1 | | 2.7 |
| - | y4047 | hypothetical protein | 3.3 | | 3.2 | | 2.2 |
|  | | | | | | | |
| *Not in COGS* | | | | | | | |
| - | y0063 | hypothetical protein | 2.9 | | 3.4 | | 3.0 |
| *yitA* | y0183 | insecticidal toxin subunit | 5.5 | | 27.1 | | 8.8 |
| *yitC* | y0185 | insecticidal toxin subunit | 11.4 | | 21.0 | | 10.0 |
| - | y0186 | putative holin protein of prophage | 11.5 | | 30.1 | | 17.5 |
| - | y0187 | putative phage-related protein | *f* | | *f* | | 7.1 |
| - | y0188 | putative phage related protein | 8.9 | | 49.2 | | 20.8 |
| - | y0189 | putative phage related protein | 10.9 | | 24.7 | | 11.4 |
| *yipB* | y0191 | insecticidal toxin subunit | 3.9 | | 10.0 | | 5.6 |
| - | y0192 | putative integral membrane protein | 3.2 | | *f* | | *f* |
| - | y0264 | hypothetical protein in putative T6SS (IAHP) locus | 2.5 | | 2.4 | | 2.2 |
| - | y0357 | hypothetical protein | 2.4 | | 5.8 | | 2.5 |
| - | y0421 | hypothetical protein | 2.0 | | 2.0 | | 2.2 |
| *pspG* | y0575 | phage shock protein G | 4.9 | | 5.6 | | 4.4 |
| - | y0915 | putative lipoprotein | 3.1 | | 2.5 | | 2.0 |
| - | y1113 | hypothetical protein | 2.6 | | 3.9 | | 5.6 |
| - | y1114 | hypothetical protein | 3.9 | | 5.6 | | 4.7 |
| - | y1160 | hypothetical protein | 2.9 | | 7.1 | | 2.9 |
| - | y1161 | hypothetical protein | *f* | | *f* | | *f* |
| - | y1169 | hypothetical protein | 2.1 | | 5.6 | | 5.6 |
| - | y1266 | hypothetical protein | 4.2 | | 7.7 | | 4.4 |
| - | y1323 | hypothetical protein | 4.4 | | 4.7 | | 3.9 |
| - | y1328 | stationary phase inducible protein CsiE | 2.1 | | 8.3 | | 2.6 |
| - | y1360 | hypothetical protein | 4.2 | | 2.8 | | 2.3 |
| - | y1453 | hypothetical protein | 3.0 | | 3.9 | | 5.8 |
| - | y1562 | hypothetical protein | 2.8 | | 3.7 | | 2.7 |
| - | y1667 | hypothetical protein | 4.5 | | 6.0 | | 5.1 |
| - | y1700 | hypothetical protein | 4.8 | | 9.5 | | 3.9 |
| - | y1737 | hypothetical protein | 2.7 | | 3.4 | | 3.6 |
| - | y1783 | hypothetical protein | 2.1 | | 4.2 | | 4.6 |
| - | y1836 | putative lipoprotein | 2.2 | | 3.0 | | 3.0 |
| - | y1880 | hypothetical protein | 3.2 | | 2.9 | | 4.5 |
| - | y2068 | hypothetical protein | 4.3 | | 4.0 | | 3.2 |
| - | y2187 | hypothetical protein | 5.2 | | 8.3 | | 4.8 |
| - | y2121 | hypothetical protein | 2.4 | | 2.6 | | 2.1 |
| - | y2129 | hypothetical protein | *f* | | 3.8 | | 3.0 |
| - | y2313 | hypothetical protein | 5.4 | | 6.6 | | 4.8 |
| - | y2315 | hypothetical protein | 6.9 | | 6.8 | | 4.4 |
| - | y2316 | hypothetical protein | 4.9 | | 7.0 | | 3.1 |
| - | y2321 | hypothetical protein | 4.7 | | 5.0 | | 5.4 |
| - | y2412 | hypothetical protein | 4.3 | | 6.3 | | 3.9 |
| - | y2437 | hypothetical protein | 5.3 | | 3.2 | | 3.5 |
| - | y2566 | hypothetical protein | 2.1 | | 3.4 | | 2.6 |
| - | y2571 | hypothetical protein | 2.3 | | 2.4 | | 2.2 |
| - | y2616 | hypothetical protein | 7.7 | | 5.5 | | 4.1 |
| *yadB* | y2786 | putative outer membrane virulence factor | *f* | | *f* | | *f* |
| - | y2812 | hypothetical protein | 2.9 | | 4.8 | | 2.1 |
| - | y2827 | hypothetical protein | 2.0 | | 4.5 | | 2.2 |
| - | y2863 | hypothetical protein | 2.8 | | 7.1 | | 4.8 |
| *rtn* | y2909 | hypothetical EAL-domain protein | 3.1 | | 3.7 | | 2.7 |
| - | y2929 | hypothetical protein | 2.4 | | 2.8 | | 3.2 |
| - | y3250 | hypothetical protein | 2.9 | | 2.2 | | 2.1 |
| - | y3289 | hypothetical protein | 6.1 | | 5.5 | | 4.4 |
| - | y3390 | hypothetical protein | 2.9 | | 2.6 | | 4.0 |
| - | y3419 | hypothetical protein, ynp siderophore locus | 2.1 | | 3.4 | | 2.8 |
| - | y3474 | hypothetical protein | 6.0 | | 3 | | 2.6 |
| - | y3576 | hypothetical protein | 2.4 | | 2.1 | | 2.5 |
| - | y3577 | hypothetical protein | 3.5 | | 3.3 | | 2.8 |
| - | y3755 | major facilitator superfamily permease | 2.6 | | 4.3 | | 4.4 |
| - | y3928 | hypothetical protein | 3.8 | | 6.5 | | 4.3 |
| - | y3929 | hypothetical protein | 2.7 | | 2.5 | | 2.3 |
| **f,*gene transcripts detected in the flea only | | | | | | | |
