# Supplementary material for: Transit through the Flea Vector Induces a Pretransmission Innate Immunity Resistance Phenotype in Yersinia pestis
Source: PLoS Pathog. 2010 Feb 26;6(2):e1000783. doi: 10.1371/journal.ppat.1000783 (PMC2829055; doi:10.1371/journal.ppat.1000783)
Supplement: Table S2 — Y. pestis genes downregulated ≥2-fold in the flea relative to all in vitro conditions. (0.14 MB DOC) [file ppat.1000783.s004.doc]

**Table S2. *Y. pestis* genes downregulated ≥ 2-fold in the flea relative to all *in vitro* conditions**

| **gene** | **orf** | **predicted function of gene product** | **fold change* relative to:** | | |
| --- | --- | --- | --- | --- | --- |
|  |  |  | **flowcell biofilm** | **exp. phase planktonic** | **stat. phase planktonic** |
| *Amino acid transport and metabolism* | | | | | |
| *ureA* | y1237 | urease gamma subunit | -21.8 | -4.5 | -3.2 |
| *ureB* | y1238 | urease beta subunit | -13.8 | -3.6 | -2.6 |
| *ureC* | y1239 | urease alpha subunit | -11.6 | -2.2 | -2.4 |
| *-* | y2137 | putative arginine/ornithine antiporter | *iv* | *iv* | *iv* |
| - | y2870 | putative hydrolase | *iv* | *iv* | *iv* |
| *gltJ* | y4124 | aspartate/glutamate ABC transport system permease | *iv* | *iv* | *iv* |
|  | | | | | |
| *Carbohydrate transport and metabolism* | | | | | |
| *lamB* | y0032 | maltoporin precursor | -5.4 | -2.6 | -4.7 |
| *manX* | y2551 | PTS system, mannose-specific IIAB component | -3.2 | -4.9 | -5.0 |
| *manZ* | y2553 | PTS system, mannose-specific IID component | -2.1 | -2.4 | -2.7 |
|  | | | | | |
| *Cell wall/membrane biogenes*i**s** | | | | | |
| *kdtX* | y0087 | lipopolysaccharide core biosynthesis glycosyl transferase | -2.2 | -2.6 | -2.0 |
| *-* | y2759 | putative outer membrane porin C protein | -2.1 | -2.8 | -2.7 |
| *mscL* | y4019 | large-conductance mechanosensitive channel | -2.4 | -6.3 | -2.2 |
|  | | | | | |
| *Defense mechanisms* | | | | | |
| *pcp* | y0221 | outer membrane lipoprotein | -2.1 | -4.6 | -2.7 |
|  | | | | | |
| *Inorganic ion transport and metabolism* | | | | | |
| *sbp1* | y0058 | exported sulfate-binding protein | *iv* | *iv* | *iv* |
| *zntA* | y0410 | zinc, lead, cadmium, and mercury transporting ATPase | *iv* | *iv* | *iv* |
| *pstb* | y1401 | putative phosphate transport ATP-binding protein | *iv* | *iv* | *iv* |
| *cysP* | y1466 | thiosulfate-binding protein | -3.8 | -2.3 | -4.0 |
| *cysA* | y1469 | sulfate transport ATP-binding protein | *iv* | *iv* | *iv* |
| *dps* | y1677 | DNA protection during starvation conditions | -3.6 | -4.2 | -4.8 |
| *wrbA* | y2449 | TrpR binding protein | -2.3 | -5.3 | -4.6 |
| *bfr* | y3988 | bacterioferritin | -2.2 | -3.5 | -2.4 |
|  | | | | | |
| *Intracellular trafficking and secretion* | | | | | |
| *ftsY* | y0416 | cell division protein | *iv* | *iv* | *iv* |
| *cysQ* | y0654 | inositol monophosphatase family protein | -2.6 | -4.7 | -2.9 |
| *secG* | y0685 | protein-export membrane protein | -2.6 | -2.0 | -2.2 |
|  | | | | | |
| *Nucleotide transport and metabolism* | | | | | |
| *codA* | y3946 | cytosine deaminase | *iv* | *iv* | *iv* |
|  | | | | | |
| *Posttranslational modification, protein turnover, chaperones* | | | | | |
| *ilvL* | y0335 | ilvGEDA operon leader peptide | -6.7 | -4.4 | -2.3 |
| *groES* | y0608 | co-chaperonin GroES | -2.3 | -2.5 | -2.2 |
| *bcp* | y1417 | bacterioferritin comigratory protein | -2.2 | -3.9 | -2.0 |
| *hslJ* | y2003 | putative heat shock protein | -2.4 | -2.4 | -3.0 |
| *grxA* | y2855 | glutaredoxin 1 | -4.7 | -2.9 | -2.4 |
|  | | | | | |
| *Replication, recombination and repair* | | | | | |
| *priC* | y1056 | primosomal replication protein n'' | *iv* | *iv* | *iv* |
| *-* | y3540 | hypothetical protein | *iv* | *iv* | *iv* |
|  | | | | | |
| *Secondary metabolites biosynthesis, transport and catabolism* | | | | | |
| *-* | y0777 | multicopper oxidase | *iv* | *iv* | *iv* |
| *trxC* | y0919 | thioredoxin 2 | -2.4 | -3.6 | -2.2 |
|  | | | | | |
| *Transcription and signal transduction mechanisms* | | | | | |
| *pmrB* | y0676 | two-component system sensor protein | *iv* | *iv* | *iv* |
| - | y0984 | short chain dehydrogenase | -2.5 | -3.4 | -3.1 |
| *rovA/slyA* | y1961 | transcriptional regulator SlyA | -7.3 | -6.5 | -5.9 |
| *psaE* | y2884 | putative regulatory protein | -4.4 | -7.9 | -20.3 |
|  | | | | | |
| *Translation* | | | | | |
| *-* | y0163 | hypothetical protein | -2.4 | -4.5 | -3.5 |
| *trmA* | y0324 | tRNA (uracil-5-)-methyltransferase | *iv* | *iv* | *iv* |
| *-* | y2240 | hypothetical protein | -2.8 | -2.0 | -2.2 |
| *infA* | y2807 | translation initiation factor IF-1 | -3.8 | -2.6 | -2.0 |
|  | | | | | |
| *General function prediction and function unknown* | | | | | |
| - | y0036 | hemolysin co-regulated protein | *iv* | *iv* | *iv* |
| - | y0045 | hypothetical protein | -2.4 | -3.1 | -2.7 |
| - | y0755 | zinc-binding protein | -2.0 | -2.1 | -3.1 |
| - | y1455 | hypothetical protein | -3.2 | -5.7 | -3.9 |
| - | y1877 | probable N-acetylmuramoyl-L-alanine amidase | -2.8 | -4.4 | -3.1 |
| - | y2529 | hypothetical protein | -2.4 | -3.1 | -2.5 |
| - | y3588 | hypothetical protein | -6.7 | -3.3 | -2.8 |
| - | y3609 | hypothetical protein | -3.8 | -4.5 | -4.5 |
| - | y3961 | hypothetical protein | -3.1 | -2.3 | -4.5 |
|  | | | | | |
| *Not in COGs* | | | | | |
| - | y0164 | hypothetical protein | -2.1 | -4.5 | -6.8 |
| - | y0288 | hypothetical protein | -2.1 | -3.4 | -7.3 |
| - | y1185 | alpha helical protein | -2.5 | -4.8 | -2.1 |
| - | y2017 | putative invertase protein (partial) | -2.3 | -3.4 | -2.5 |
| - | y3636 | hypothetical protein | -3.0 | -2.3 | -7.3 |
| **iv,* gene transcripts detected *in vitro* only | | | | | |
