# Supplementary material for: Transit through the Flea Vector Induces a Pretransmission Innate Immunity Resistance Phenotype in Yersinia pestis
Source: PLoS Pathog. 2010 Feb 26;6(2):e1000783. doi: 10.1371/journal.ppat.1000783 (PMC2829055; doi:10.1371/journal.ppat.1000783)
Supplement: Table S3 — The 100 most highly expressed Y. pestis genes in the flea. (0.22 MB DOC) [file ppat.1000783.s005.doc]

**Table S3. The 100 most highly expressed *Y. pestis* genes in the flea**

| **gene** | **orf** | **predicted function of gene product** | **relative expression*** | **rank** |
| --- | --- | --- | --- | --- |
| **A. Plasmid genes** | | |  |  |
| *pla* | ypkp07 | coagulase/fibrinolysin precursor | 25.0 | 9 |
| *ymt* | y1069 | murine toxin | 12.9 | 27 |
|  | | |  |  |
| **B. Chromosomal genes** | | |  |  |
| *Amino acid transport and metabolism* | | | | |
| *hutI* | y2340 | imidazolonepropionase | 7.2 | 71 |
| *aspC2* | y3555 | aspartate aminotransferase | 27.6 | 7 |
| *sdaC* | y2862 | serine transporter | 12.2 | 31 |
| *-* | y3595 | putative symporter protein | 8.6 | 52 |
| *glnH* | y1189 | putative amino acid-binding protein precursor | 6.8 | 76 |
| *Carbohydrate transport and metabolism* | | | | |
| *-* | y3770 | putative ABC transporter permease protein | 6.4 | 84 |
| *spr* | y2908 | putative lipoprotein | 15.1 | 19 |
| *lpxC* | y3620 | UDP-3-O-[3-hydroxymyristoyl] N-acetylglucosamine deacetylase | 8.8 | 48 |
| *ompA* | y2735 | putative outer membrane porin A protein | 7.4 | 67 |
| *ail* | y1324 | attachment invasion locus protein | 7.3 | 69 |
| *-* | y2759 | putative outer membrane porin C protein | 6.7 | 78 |
| *mraW* | y3634 | S-adenosyl-methyltransferase | 6.0 | 90 |
| *lpp* | -*^a^* | major outer membrane protein | 13.1 | 26 |
| *Coenzyme transport and metabolism* | | | | |
| *cyoE* | y1018 | protoheme IX farnesyltransferase | 14.4 | 21 |
| *menG* | y0292 | ribonuclease activity regulator protein | 7.8 | 61 |
| *ribB* | y3520 | 3,4-dihydroxy-2-butanone 4-phosphate synthase | 5.8 | 100 |
| *Energy production and conversion* | | | | |
| *sdhC* | y3071 | succinate dehydrogenase cytochrome b556 large membrane subunit | 34.6 | 6 |
| *sdhD* | y3070 | succinate dehydrogenase cytochrome b556 small membrane subunit | 12.4 | 29 |
| *sdhB* | y3068 | succinate dehydrogenase catalytic subunit | 6.1 | 86 |
| *sucB* | y3066 | dihydrolipoamide acetyltransferase | 6.0 | 92 |
| *atpE* | y4140 | ATP synthase subunit C | 15.4 | 17 |
| *atpB* | y4141 | ATP synthase subunit A | 7.0 | 72 |
| *atpI* | y4142 | ATP synthase subunit I | 8.9 | 47 |
| *frdD* | y0614 | fumarate reductase subunit D | 11.2 | 34 |
| *nqrE* | y0955 | Na(+)-translocating NADH-quinone reductase subunit E | 6.4 | 82 |
| *nuoA* | y1630 | NADH dehydrogenase alpha subunit | 6.4 | 83 |
| *napF* | y1442 | ferredoxin-type protein NapF | 5.9 | 97 |
| *Inorganic ion transport and metabolism* | | | | |
| *ftnA* | y2524 | ferritin | 7.4 | 68 |
| *secY* | y4010 | preprotein translocase | 12.4 | 30 |
| *secE* | y0478 | translocase | 6.7 | 77 |
| *Lipid transport and metabolism* | | | | |
| *acpP* | y1759 | acyl carrier protein | 10.7 | 37 |
|  | | | | |
| *Posttranslational modification, protein turnover, chaperones* | | | | |
| *trxA* | y0360 | thioredoxin 1 | 6.0 | 88 |
| *Replication, recombination and repair* | | | | |
| *ihfB* | y2779 | integration host factor beta subunit | 11.7 | 32 |
| *hupA* | y0499 | DNA-binding protein HU-alpha | 10.7 | 38 |
| *tnp* | y0044 | transposase for the IS1541 insertion element | 9.0 | 46 |
| *Signal transduction mechanisms* | | | | |
| *rseA* | y1291 | sigma E factor negative regulatory protein | 13.8 | 24 |
| *rseB* | y1292 | periplasmic negative regulator of sigmaE | 8.6 | 54 |
| *uspA* | y3859 | universal stress protein A | 10.9 | 35 |
| *phoP* | y1794 | response regulator protein | 9.1 | 44 |
| *sixA* | y1582 | putative phosphohistidine phosphatase | 7.8 | 60 |
| *csrA* | y0884 | carbon storage regulator | 5.9 | 94 |
| *fimZ* | y2386 | fimbrial Z protein signal transducer | 5.9 | 98 |
| *Transcription* | | | | |
| *cspE* | y1166 | cold shock protein E | 38.1 | 5 |
| *cspa1* | y0223 | major cold shock protein Cspa1 | 26.3 | 8 |
| *cspa2* | y0224 | major cold shock protein Cspa2 | 10.5 | 39 |
| *rpoS* | y0834 | RNA polymerase sigma factor | 9.3 | 42 |
| *rpoE* | y1290 | RNA polymerase sigma-70 factor | 7.9 | 58 |
| *-* | y3766 | hypothetical protein | 7.7 | 63 |
| *yitR* | y0181 | putative lysR-family transcriptional regulatory protein | 6.1 | 87 |
| *-* | y3765 | *sorC* family transcriptional regulator | 8.8 | 49 |
| *Translation* | | | | |
| *rpsO* | y0691 | 30S ribosomal protein S15 | 45.2 | 1 |
| *rpmH* | y4114 | 50S ribosomal protein L34 | 45.0 | 2 |
| *rpsU* | y3535 | 30S ribosomal protein S21 | 44.9 | 3 |
| *infC* | y1904 | translation initiation factor IF-3 | 22.4 | 10 |
| *rplT* | y1905 | 50S ribosomal protein L20 | 19.0 | 13 |
| *rplL* | y0483 | 50S ribosomal protein L7/L12 | 6.0 | 91 |
| *rplJ* | y0482 | 50S ribosomal protein L10 | 16.0 | 15 |
| *rplA* | y0481 | 50S ribosomal protein L1 | 15.6 | 16 |
| *-* | y1753 | 50S ribosomal protein L32 | 14.1 | 23 |
| *rplN* | y3999 | 50S ribosomal protein L14 | 6.0 | 93 |
| *rplE* | y4001 | 50S ribosomal protein L5 | 16.9 | 14 |
| *rpsH* | y4003 | 30S ribosomal protein S8 | 12.6 | 28 |
| *rplF* | y4004 | 50S ribosomal protein L6 | 10.4 | 40 |
| *rpmD* | y4008 | 50S ribosomal protein L30 | 13.6 | 25 |
| *rplO* | y4009 | 50S ribosomal protein L15 | 5.9 | 99 |
| *rpsM* | y4012 | 30S ribosomal protein S13 | 19.5 | 12 |
| *rpsK* | y4013 | 30S ribosomal protein S11 | 7.6 | 65 |
| *rpsD* | y4014 | 30S ribosomal protein S4 | 10.9 | 36 |
| *-* | y3550 | putative translational inhibitor protein | 9.2 | 43 |
| *rplU* | y0672 | 50S ribosomal protein L21 | 8.3 | 55 |
| *rpmA* | y0673 | 50S ribosomal protein L27 | 11.4 | 33 |
| *rpsP* | y0894 | 30S ribosomal protein S16 | 7.4 | 66 |
| *serS* | y2796 | seryl-tRNA synthetase | 6.8 | 75 |
| *rplY* | y2920 | 50S ribosomal protein L25 | 6.4 | 81 |
| *General function prediction and function unknown* | | | | |
| *-* | y1752 | hypothetical protein | 9.0 | 45 |
| *-* | y2523 | putative copper resistance protein | 8.2 | 56 |
| *hfq* | y0630 | RNA-binding protein Hfq | 8.1 | 57 |
| *-* | y2260 | hypothetical protein | 7.2 | 70 |
| *-* | y0708 | putative acetyltransferase | 6.6 | 79 |
| *-* | y3609 | hypothetical protein | 7.7 | 62 |
| *-* | y3610 | hypothetical protein | 14.2 | 22 |
| *-* | y3060 | hypothetical protein | 7.9 | 59 |
| *-* | y3774 | hypothetical protein | 6.9 | 73 |
| *-* | y3635 | hypothetical protein | 5.9 | 96 |
| *Not in COGs* | | | | |
| *ilvB* | y2125 | ilvB operon leader peptide | 39.2 | 4 |
| *-* | y2296 | hypothetical protein | 20.4 | 11 |
| *-* | y3038 | hypothetical protein | 15.1 | 18 |
| *-* | y1160 | hypothetical protein | 15.1 | 20 |
| *-* | y1783 | hypothetical protein | 10.4 | 41 |
| *-* | y2914 | hypothetical protein | 8.7 | 50 |
| *-* | y0826 | hypothetical protein | 8.6 | 51 |
| *hisL* | y2618 | his operon leader peptide | 8.6 | 53 |
| *uspB* | y3860 | universal stress protein | 7.7 | 64 |
| *-* | y3061 | hypothetical protein | 6.9 | 74 |
| *-* | y2193 | hypothetical protein | 6.5 | 80 |
| *-* | y0323 | hypothetical protein | 6.1 | 89 |
| *-* | y1486 | hypothetical protein | 5.9 | 95 |
| *-* | y2986 | hypothetical protein | 6.3 | 85 |
| *, hybridization signal of the indicated gene divided by the average signal of all 4,683 *Y. pestis* genes on the microarray (samples from flea only)  *^a^****,*** gene not annotated in *Y. pestis* KIM6+; gene annotated as YPO2394 in *Y. pestis* CO92 | | | | |
