# Supplementary material for: Transit through the Flea Vector Induces a Pretransmission Innate Immunity Resistance Phenotype in Yersinia pestis
Source: PLoS Pathog. 2010 Feb 26;6(2):e1000783. doi: 10.1371/journal.ppat.1000783 (PMC2829055; doi:10.1371/journal.ppat.1000783)
Supplement: Table S4 — Y. pestis genes upregulated ≥2-fold in the flea and flowcell biofilms relative to planktonic culture conditions. (0.12 MB DOC) [file ppat.1000783.s006.doc]

**Table S4.** *Y. pestis* genes upregulated ≥ 2-fold in the flea and flowcell biofilms relative to planktonic culture conditions

| **gene** | **orf** | **predicted function of gene product** | **fold change (flea)**  **relative to:** | | **fold change (flowcell) relative to:** | |
| --- | --- | --- | --- | --- | --- | --- |
|  |  |  | **exp.** | **stat.** | **exp.** | **stat.** |
| *Amino acid transport and metabolism* | | | | | | |
| *glpB* | y0405 | anaerobic glycerol-3-phosphate dehydrogenase subunit B | 3.6 | 2.8 | 13.5 | 10.5 |
| *pepT* | y1791 | peptidase T | 3.9 | 2.9 | 6.6 | 5.0 |
| *artM* | y2832 | arginine transport system permease protein | 4.0 | 3.8 | 2.7 | 2.5 |
|  | | | | | | |
| *Carbohydrate transport and metabolism* | | | | | | |
| *glpF* | y0046 | glycerol uptake facilitator protein | 6.2 | 4.3 | 18.9 | 13.2 |
| *gpmM* | y0077 | phosphoglyceromutase | 2.3 | 2.2 | 3.7 | 3.6 |
| *galK* | y3045 | galactokinase | 2.6 | 3.1 | 2.3 | 2.7 |
|  | | | | | | |
| *Cell wall/membrane biogenesis* | | | | | | |
| *-* | y0545 | hypothetical protein | 2.0 | 2.1 | 3.3 | 3.4 |
| *-* | y2044 | hypothetical protein | 24.9 | 21.9 | 3.8 | 3.4 |
|  | | | | | | |
| *Coenzyme transport and metabolism* | | | | | | |
| *-* | y0547 | coproporphyrinogen III oxidase | 3.2 | 2.8 | 4.0 | 3.5 |
| *menF* | y1659 | menaquinone-specific isochorismate synthase | 4.5 | 4.5 | 3.7 | 3.7 |
| *menD* | y1660 | 2-oxoglutarate decarboxylase | 2.4 | 2.5 | 4.0 | 4.2 |
| *bioD* | y2111 | putative dethiobiotin synthetase | 2.3 | 2.1 | 9.5 | 8.7 |
|  | | | | | | |
| *Energy production and conversion* | | | | | | |
| *glpC* | y0404 | anaerobic glycerol-3-phosphate dehydrogenase subunit A | 5.3 | 4.3 | 28.8 | 23.2 |
| *glpA* | y0406 | anaerobic glycerol-3-phosphate dehydrogenase subunit C | 6.2 | 4.5 | 15.4 | 11.1 |
| *gltP* | y0511 | proton glutamate symport protein | 4.0 | 3.1 | 2.7 | 2.1 |
| *dmsB* | y0865 | anaerobic dimethyl sulfoxide reductase chain B | 8.5 | 8.6 | 14.8 | 15.1 |
| *-* | y1442 | ferredoxin-type protein NapF | 2.2 | 4.9 | 3.7 | 8.0 |
| *napC* | y1447 | cytochrome C-type protein NapC | 2.6 | 3.1 | 4.5 | 5.4 |
| *glpD* | y3891 | aerobic glycerol-3-phosphate dehydrogenase (partial) | 5.1 | 2.7 | 14.9 | 7.9 |
|  | | | | | | |
| *Inorganic ion transport and metabolism* | | | | | | |
| *-* | y0546 | hypothetical protein | 2.6 | 3.3 | 3.5 | 4.5 |
| *-* | y1443 | hypothetical protein | 2.2 | 5.2 | 3.9 | 9.1 |
| *-* | y1626 | putative ion transport protein | 2.5 | 3.2 | 3.5 | 4.5 |
| *fcuA* | y2556 | ferrichrome receptor protein | 2.9 | 2.3 | 2.7 | 2.1 |
|  | | | | | | |
| *Intracellular trafficking and secretion* | | | | | | |
| *cpxP* | y0066 | periplasmic protein | 3.1 | 3.4 | 2.9 | 3.1 |
|  | | | | | | |
| *Lipid transport and metabolism* | | | | | | |
| *-* | y1115 | putative CDP-alcohol phosphatidyltransferase | 3.9 | 3.7 | 2.1 | 2.0 |
| *-* | y1117 | putative acyltransferase | 5.0 | 5.7 | 2.3 | 2.6 |
|  | | | | | | |
| *Nucleotide transport* | | | | | | |
| *nrdD* | y0733 | anaerobic ribonucleoside triphosphate reductase | 2.2 | 7.1 | 6.0 | 19.5 |
|  | | | | | | |
| *Posttranslational modification, protein turnover, chaperones* | | | | | | |
| *yhbU* | y0706 | putative protease | 2.4 | 2.8 | 4.6 | 5.3 |
| *ccmH* | y1574 | putative cytochrome c-type biogenesis protein | 2.0 | 4.2 | 4.4 | 9.3 |
| *-* | y1575 | putative cytochrome c-type biogenesis protein | 2.0 | 2.3 | 3.4 | 3.9 |
|  | | | | | | |
| *Transcription* | | | | | | |
| *yitR* | y0181 | putative lysR-family transcriptional regulator | 47.2 | 23.4 | 4.6 | 2.2 |
| *terZ* | y0555 | tellurium resistance protein | 2.2 | 2.0 | 7.3 | 6.7 |
|  | | | | | | |
| *Translation* | | | | | | |
| *hemK* | y2289 | N5-glutamine S-adenosyl-L-methionine-dependent methyltransferase | 2.8 | 2.7 | 2.1 | 2.0 |
|  | | | | | | |
| *General function prediction and function unknown* | | | | | | |
| *dcuA* | y0605 | anaerobic C4-dicarboxylate transporter | 3.4 | 2.4 | 3.3 | 2.3 |
| *dmsC* | y0866 | anaerobic dimethyl sulfoxide reductase chain C | 6.4 | 5.2 | 9.7 | 8.0 |
| *-* | y0867 | hypothetical protein | 5.5 | 6.6 | 9.1 | 10.9 |
| *-* | y1116 | putative phosphatidate cytidylyltransferase | 3.9 | 3.4 | 2.6 | 2.2 |
| *yfiD* | y1282 | hypothetical protein | 3.4 | 2.7 | 9.7 | 7.7 |
| *dmsC* | y1517 | putative dimethyl sulfoxide reductase chain C protein | 2.4 | 2.2 | 3.1 | 2.8 |
|  | y3707 | hypothetical protein | 4.1 | 3.5 | 5.5 | 4.7 |
| *eptB* | y4034 | LPS-phosphoethanolamine transferase | 4.3 | 4.2 | 3.3 | 3.3 |
| *yidE* | y4100 | hypothetical membrane protein | 2.5 | 5.2 | 2.0 | 4.3 |
|  | | | | | | |
| *Not in COGS* | | | | | | |
|  | y0188 | hypothetical protein | 49.2 | 20.8 | 5.5 | 2.3 |
|  | y1267 | hypothetical protein | 10.6 | 3.3 | 9.7 | 3.0 |
|  | y1901 | hypothetical protein | *ns* | 3.7 | 3.9 | 2.7 |
|  | y2296 | hypothetical protein | 4.7 | 2.6 | 3.9 | 2.1 |
|  | y2770 | hypothetical protein | 2.8 | 2.6 | 4.0 | 3.7 |
