# Supplementary material for: Transit through the Flea Vector Induces a Pretransmission Innate Immunity Resistance Phenotype in Yersinia pestis
Source: PLoS Pathog. 2010 Feb 26;6(2):e1000783. doi: 10.1371/journal.ppat.1000783 (PMC2829055; doi:10.1371/journal.ppat.1000783)
Supplement: Table S5 — Y. pestis genes with significantly higher relative expression levels in the flea gut than in the rat bubo. (0.28 MB DOC) [file ppat.1000783.s007.doc]

Table S5. *Y. pestis* genes with significantly higher relative expression levels in the flea gut than in the rat bubo

| **gene** | **KIM orf** | **predicted function of gene product** | **CO92 orf** | **relative expression***  **(bubo/flea)** |
| --- | --- | --- | --- | --- |
| **A. Plasmid genes** | | | | |
| *pim* | Ypkp04 | pesticin immunity protein | YpPCP1.03 | -6.6 |
|  | | | | |
| **B. Chromosomal genes** | | | | |
| *Amino acid transport and metabolism* | | | | |
| *-* | y0838 | dehydrogenase | YPO3352 | -4.8 |
| *-* | y0932 | solute-binding protein of ABC transporter | YPO3257 | -6.2 |
| *-* | y0933 | inner membrane permease of ABC transporter | YPO3256 | *f* |
| *-* | y0934 | putative amino acid ABC transporter, permease protein | YPO3255 | *f* |
| *gltL* | y1186 | ATP-binding protein of glutamate/aspartate transport system | YPO2612 | -4.9 |
| *gltK* | y1187 | glutamate/aspartate inner membrane permease | YPO2613 | -6.6 |
| *gltJ* | y1188 | glutamate/aspartate ABC transport system inner membrane permease | YPO2614 | -4.1 |
| *-* | y1233 | permease of ABC transporter | YPO2662 | *f* |
| *gabT* | y1390 | 4-aminobutyrate aminotransferase | YPO2844 | *f* |
| *potD* | y1391 | substrate-binding protein of ABC transporter | YPO2843 | -12.6 |
| *potB* | y1392 | permease of ABC transporter | YPO2842 | *f* |
| *potC* | y1393 | permease of ABC transporter | YPO2841 | -4.7 |
| *hisQ* | y1608 | histidine ABC transport system inner membrane permease | YPO2775 | *f* |
| *glnP* | y1674 | glutamine ABC transporter permease protein | YPO2513 | -8.1 |
| *glnH* | y1675 | glutamine ABC transporter periplasmic protein | YPO2512 | -4.1 |
| *hutI* | y2340 | imidazolonepropionase | YPO1972 | -26.4 |
| *hutG* | y2341 | histidine degradation enzyme | YPO1971 | -14.1 |
| *astA* | y2348 | arginine succinyltransferase | YPO1963 | -5.9 |
| *ansB* | y2787 | L-asparaginase II | YPO1386 | -5.3 |
| *artP* | y2829 | arginine transporter ATP-binding subunit | YPO1352 | -4.8 |
| *artQ* | y2831 | arginine transporter permease subunit | YPO1350 | -4.3 |
| *gabD* | y2894 | succinate-semialdehyde dehydrogenase | YPO1290 | -5.1 |
| *aspC* | y3555 | aspartate aminotransferase | YPO0623 | -18.5 |
| *-* | y3595 | Na/dicarboxylate symporter | YPO0584 | -4.0 |
| *dppB* | y3826 | dipeptide transport system inner membrane permease | YPO4002 | -5.8 |
| *hutH* | y4037 | histidine ammonia-lyase | YPO4016 | -8.8 |
| *hutU* | y4038 | urocanate hydratase | YPO4017 | -5.1 |
|  | | | | |
| *Carbohydrate transport and metabolism* | | | | |
| *glpF* | y0046 | facilitator for glycerol uptake | YPO0091 | -4.6 |
| *-* | y0329 | putative sugar transport system ATP-binding protein | YPO3907 | *f* |
| *-* | y0674 | hypothetical protein | YPO3510 | -4.8 |
| *-* | y0773 | hypothetical protein | YPO3413 | -4.5 |
| *-* | y0926 | probable sugar transporter | YPO3263 | *f* |
| *chbB* | y1251 | chitobiose PTS system transporter subunit IIB | YPO2678 | -4.3 |
| *-* | y1595 | hypothetical protein | YPO2761 | *f* |
| *araH* | y2098 | L-arabinose transporter permease protein | YPO2257 | *f* |
| *-* | y2586 | hypothetical protein | YPO1578 | -8.0 |
| *-* | y2587 | membrane permease of ABC transporter | YPO1577 | -6.8 |
| *mglC* | y2660 | beta-methylgalactoside transporter inner membrane component | YPO1509 | -5.8 |
| *-* | y2961 | oligogalacturonide transporter | YPO1226 | *f* |
| *-* | y3770 | ABC transport system permease protein | YPO0411 | -6.9 |
| *frwC* | y3777 | PTS system fructose-like enzyme 2IIC component | YPO0404 | *f* |
| *frwD* | y3780 | PTS system fructose-like IIB component 2 | YPO0402 | *f* |
|  | | | | |
| *Cell cycle control, mitosis and meiosis* | | | | |
| *ccrB* | y1168 | camphor resistance protein CrcB | YPO2596 | -6.5 |
| *-* | y2040 | intracellular septation protein A | YPO2196 | -4.7 |
|  | | | | |
| *Cell motility* | | | | |
|  | y3170 | prepilin peptidase protein | YPO1014a | *f* |
|  | | | | |
| *Cell wall/membrane biogenesis* | | | | |
| *-* | y2825 | nucleotide di-P-sugar epimerase or dehydratase | YPO1355 | -5.2 |
| *spr* | y2908 | lipoprotein | YPO1275 | -4.6 |
| *-* | y3968 | putative glycosyl transferase | YPO0187 | -8.5 |
|  | | | | |
| *Coenzyme transport and metabolism* | | | | |
| *cyoE* | y1018 | protoheme IX farnesyltransferase | YPO3168 | -5.6 |
| *ascD* | y1067 | CDP-6-deoxy-delta-3,4-glucoseen reductase | YPO3116 | *f* |
|  | | | | |
| *Defense mechanisms* | | | | |
| *-* | y3423 | ATP-binding protein | YPO0770 | *f* |
|  | | | | |
| *Energy production and conversion* | | | | |
| *frdD* | y0614 | fumarate reductase subunit D | YPO0357 | -4.5 |
| *-* | y0700 | hypothetical protein | YPO3484 | -3.9 |
| *-* | y0955 | Na(+)-translocating NADH-quinone reductase subunit E | YPO3236 | -5.2 |
| *sdhD* | y3070 | succinate dehydrogenase cytochrome b556 small membrane subunit | YPO1110 | -5.7 |
| *sdhC* | y3071 | succinate dehydrogenase cytochrome b556 large membrane subunit | YPO1109 | -9.7 |
| *nhaC* | y3554 | putative Na+/H+ antiporter | YPO0624 | -18.8 |
| *dctA* | y3836 | C4-dicarboxylate symporter DctA | YPO3992 | -6.8 |
| *atpI* | y4142 | F0F1 ATP synthase subunit I | YPO4128 | -7.6 |
|  |  |  |  |  |
| *Extracellular structures* | | | | |
| *-* | y4063 | fimbrial-like protein | YPO4044 | *f* |
|  | | | | |
| *Inorganic ion transport and metabolism* | | | | |
| *emrE* | y2000 | methylviologen resistance | YPO2333 | -6.2 |
| *clcB* | y2112 | putative voltage-gated ClC-type chloride channel ClcB | YPO2270 | *f* |
| *mdtI* | y2241 | multidrug efflux system protein MdtI | YPO2069 | -4.6 |
|  | | | | |
| *Intracellular trafficking and secretion* | | | | |
| *tatE* | y1170 | twin arginine translocation protein E | YPO2597 | -5.6 |
| *-* | y3169 | prepilin peptidase-dependent protein | YPO1015 | -14.5 |
|  | | | | |
| *Lipid transport and metabolism* | | | | |
| *-* | y2039 | predicted hydrolase | YPO2195 | -3.9 |
| *-* | y2859 | undecaprenyl pyrophosphate phosphatase | YPO1324 | -9.3 |
| *cdsA* | y3129 | CDP-diglyceride synthase | YPO1050 | -4.0 |
|  | | | | |
|  | | | | |
| *Nucleotide transport and metabolism* | | | | |
| *-* | y2338 | N-formimino-L-glutamate deiminase | YPO1974 | -5.2 |
|  | | | | |
| *Posttranslational modification, protein turnover, chaperones* | | | | |
| *ccmB* | y1568 | heme exporter protein B | YPO2735 | -5.4 |
|  | | | | |
| *Replication, recombination and repair* | | | | |
| *-* | y0240 | DNA-damage inducible protein | YPO3628 | -4.8 |
| *-* | y1213 | hypothetical protein | YPO2639 | -4.4 |
| *recO* | y1299 | DNA repair protein RecO | YPO2720 | -5.3 |
|  | | | | |
| *Secondary metabolites biosynthesis, transport and catabolism* | | | | |
| *-* | y2599 | 2-hydroxyhepta-2,4-diene-1, 7-dioateisomerase / | YPO1566 | -4.4 |
|  |  | 5-carboxymethyl-2-oxo-hex-3-ene-1, 7-dioatedecarboxylase |  |  |
|  | y2600 | oxidoreductase | YPO1565 | -16.0 |
|  | | | | |
| *Signal transduction mechanisms* | | | | |
| *-* | y1582 | phosphohistidine phosphatase | YPO2748 | -4.4 |
| *hnr* | y2148 | response regulator of RpoS | YPO2173 | -4.9 |
| *fimZ* | y2386 | fimbrial Z protein; signal transducer | YPO1925 | -6.1 |
| *uhpA* | y2588 | response regulator | YPO1576 | *f* |
| *rtn* | y2909 | hypothetical EAL-domain protein | YPO1274 | *f* |
| *rcsB* | y2970 | transcriptional regulator in Rcs regulatory system | YPO1218 | -6.1 |
|  | | | | |
| *Transcription* | | | | |
| *yitR* | y0181 | transcriptional regulator | YPO3682 | *f* |
| *cspI* | y0223 | cold shock-like protein | YPO3644 | -8.6 |
| *sfsB* | y1140 | regulator | YPO2785 | -4.3 |
| *rovM* | y1629 | LysR family transcriptional regulator | YPO2556 | -11.1 |
| *rcsA* | y1741 | putative LuxR-family regulator in Rcs regulatory system | YPO2449 | -9.0 |
| *-* | y2570 | AraC-family transcriptional regulatory protein | YPO1737 | *f* |
|  | | | | |
| *Translation* | | | | |
| *-* | y0680 | hypothetical protein | YPO3504 | -4.2 |
| *rpsU* | y3535 | 30S ribosomal protein S21 | YPO0645 | -6.2 |
| *-* | y3550 | hypothetical protein | YPO0628 | *f* |
| *-* | y3551 | putative translational inhibitor protein | YPO0627 | -23.7 |
| *rpmH* | y4114 | 50S ribosomal protein L34 | YPO4100 | -7.7 |
|  | | | | |
| *General function prediction and function unknown* | | | | |
| *wzxE* | y0370 | cytochrome | YPO3858 | -5.3 |
| *-* | y0411 | enzyme | YPO3819 | -4.5 |
| *actP* | y0508 | acetate permease | YPO0251 | -6.3 |
| *-* | y0509 | putative inner membrane protein | YPO0252 | -6.4 |
| *-* | y0891 | hypothetical protein | YPO3297 | -5.2 |
| *-* | y0913 | hypothetical protein | YPO3276 | -4.7 |
| *-* | y1130 | hypothetical protein | YPO2801 | *f* |
| *iolB* | y1153 | myo-inositol catabolism protein iolB | YPO2585 | *f* |
| *-* | y1163 | hypothetical protein | YPO2592 | *f* |
| *-* | y1260 | hypothetical protein | YPO2687 | -4.1 |
| *-* | y1429 | hypothetical protein | YPO3051 | -4.2 |
| *-* | y1517 | anchor protein for oxidoreductase | YPO2967 | *f* |
| *-* | y1676 | hypothetical protein | YPO2511 | -4.6 |
| *-* | y1698 | hypothetical protein | YPO2489 | -4.3 |
| *mgtC* | y1820 | modular of P-type ATPase | YPO1660 | -10.3 |
| *-* | y2032 | dsDNA-mimic protein | YPO2187 | -4.1 |
| *-* | y2066 | hypothetical protein | YPO2224 | -7.8 |
| *-* | y2102 | cytochrome oxidase | YPO2260 | *f* |
| *-* | y2260 | hypothetical protein | YPO2051 | -7.3 |
| *-* | y2337 | hypothetical protein | YPO1975 | -6.6 |
| *-* | y2444 | hypothetical GlpM-family inner membrane protein | YPO1863 | -17.5 |
| *-* | y2858 | hypothetical protein | YPO1325 | -7.3 |
| *-* | y2878 | hypothetical protein | YPO1307 | -5.0 |
| *-* | y2985 | hypothetical protein | YPO1203 | *f* |
| *-* | y3060 | hypothetical protein | YPO1119 | -8.6 |
| *-* | y3497 | hypothetical protein | YPO0680 | -5.2 |
| *mdaB* | y3509 | modulator of drug activity B | YPO0670 | -4.4 |
| *-* | y3610 | hypothetical protein | YPO0569a | -5.0 |
| *-* | y3654 | hypothetical protein | YPO0519 | -5.0 |
| *-* | y3689 | hypothetical protein | YPO0485 | -4.2 |
| *rbn* | y3801 | ribonuclease BN | YPO0028 | -4.7 |
| *-* | y4047 | hypothetical protein | YPO4026 | -4.8 |
|  | | | | |
| *Not in COGs* | | | | |
| *-* | y0179 | hypothetical protein | YPO3684 | -7.0 |
| *yitC* | y0185 | insecticidal toxin subunit | YPO3678 | -16.1 |
| *-* | y0186 | putative holin protein of prophage | YPO3677 | *f* |
| *-* | y0187 | putative phage related protein | YPO3676 | *f* |
| *-* | y0188 | putative phage related protein | YPO3675 | -12.5 |
| *-* | y0323 | hypothetical protein | YPO3912 | -4.8 |
| *yjfF* | y0331 | inner membrane ABC transporter permease protein | YPO3905 | -4.9 |
| *-* | y0421 | putative acetyltransferase | YPO3809 | *f* |
| *-* | y0565 | hypothetical protein | YPO0306 | -4.7 |
| *pspG* | y0575 | phage shock protein G | YPO0318 | -8.6 |
| *-* | y0776 | hypothetical protein | YPO3410 | -4.0 |
| *-* | y0826 | predicted cytochrome oxidase subunit | YPO3363 | -26.5 |
| *hha/ymoA* | y1046 | histone-like hemolysin expression-modulating protein | YPO3138 | -4.4 |
| *-* | y1074 | O-unit flippase-like protein | YPO3110 | *f* |
| *-* | y1113 | hypothetical protein | YPO2818 | -5.2 |
| *-* | y1160 | hypothetical protein | YPO2590 | -8.5 |
| *-* | y1227 | hypothetical protein | YPO2653 | -14.6 |
| *-* | y1228 | hypothetical protein | YPO2654 | *f* |
| *-* | y1323 | hypothetical protein | YPO2906 | *f* |
| *-* | y1566 | hypothetical protein | YPO2733 | -4.1 |
| *-* | y1737 | hypothetical protein | YPO2453 | -4.8 |
| *-* | y1795 | putative lipoprotein | YPO1635 | -4.5 |
| *-* | y1860 | hypothetical protein | YPO1698 | -5.3 |
| *-* | y1861 | hypothetical protein | YPO1699 | -5.5 |
| *-* | y1862 | hypothetical protein | YPO1700 | -5.1 |
| *pmrL* | y1922 | hypothetical protein | YPO2417 | -4.1 |
| *pmrM* | y1923 | hypothetical protein | YPO2416 | -6.3 |
| *-* | y1970 | hypothetical protein | YPO2362 | -9.1 |
| *-* | y2170 | cobalt-nickel resistance (export) protein | YPO2151 | -10.8 |
| *-* | y2296 | predicted inner membrane protein | YPO2012 | -4.0 |
| *-* | y2354 | hypothetical protein | YPO1956 | -4.6 |
| *-* | y2412 | hypothetical protein | YPO1897 | *f* |
| *-* | y2445 | hypothetical protein | YPO1862 | -6.7 |
| *fliZ* | y2463 | protein FliZ | YPO1844 | *f* |
| *fliT* | y2468 | flagellar biosynthesis protein FliT | YPO1839 | -3.9 |
| *-* | y2567 | hypothetical protein | YPO1740 | -5.7 |
| *-* | y2571 | hypothetical protein | YPO1736 | -11.1 |
| *-* | y2591 | hypothetical protein | YPO1573 | -4.7 |
| *-* | y2592 | inner membrane permease | YPO1572 | -6.9 |
| *-* | y2602 | transport protein | YPO1563 | -4.0 |
| *-* | y2616 | hypothetical protein | YPO1552 | -14.5 |
| *-* | y2905 | hypothetical protein | YPO1278 | -4.3 |
| *-* | y2914 | hypothetical protein | YPO1269 | -7.4 |
| *-* | y3038 | hypothetical protein | YPO1144 | -5.3 |
| *-* | y3168 | hypothetical protein | YPO1016 | -5.8 |
| *-* | y3178 | hypothetical protein | YPO0791 | -4.1 |
| *-* | y3539 | hypothetical protein | YPO0642 | *f* |
| *-* | y3553 | hypothetical protein | YPO0625 | *f* |
| *-* | y3557 | hypothetical protein | YPO0620 | -9.5 |
| *-* | y3572 | hypothetical protein | YPO0607 | *f* |
| *-* | y3761 | hypothetical protein | YPO0419 | -4.4 |
| *uspB* | y3860 | universal stress protein UspB | YPO3969 | -8.7 |
| *-* | y3878 | hypothetical protein | YPO3951 | -5.4 |
| *-* | y3958 | hypothetical protein | YPO0177 | -5.2 |
| *-* | y4052 | integrase | YPO4033 | -4.6 |
| * *f,*gene transcripts detected in the flea only | | | | |
