# Supplementary material for: Transit through the Flea Vector Induces a Pretransmission Innate Immunity Resistance Phenotype in Yersinia pestis
Source: PLoS Pathog. 2010 Feb 26;6(2):e1000783. doi: 10.1371/journal.ppat.1000783 (PMC2829055; doi:10.1371/journal.ppat.1000783)
Supplement: Table S6 — Y. pestis genes with significantly higher relative expression levels in the rat bubo than in the flea. (0.51 MB DOC) [file ppat.1000783.s008.doc]

Table S6. *Y. pestis* genes with significantly higher relative expression levels in the rat bubo than in the flea

| **gene** | **KIM orf** | **predicted function of gene product** | **CO92 orf** | **relative expression***  **(bubo/flea)** |
| --- | --- | --- | --- | --- |
| **A. Plasmid genes** | | | | |
| *-* | Y1004 | putative periplasmic protein | YpMT1.42 | *b* |
| *-* | Y1010 | hypothetical protein | YpMT1.38 | *b* |
| *-* | Y1012.S | hypothetical protein | YpMT1.36 | *b* |
| *-* | Y1015 | hypothetical protein | YpMT1.35 | *b* |
| *-* | Y1020 | putative lipoprotein | YpMT1.32 | 6.5 |
| *-* | Y1039 | hypothetical protein | YpMT1.16 | *b* |
| *caf1M* | Y1098 | putative F1 chaperone protein | YpMT1.82 | 20.4 |
| *caf1A* | Y1099 | F1 capsule anchoring protein | YpMT1.83 | 35.7 |
| *caf1* | Y1100 | F1 capsule antigen | YpMT1.84 | 42.2 |
| *-* | Y1107 | hypothetical protein | YpMT1.55 | *b* |
| *rop* | Ypkp03 | putative replication regulatory protein | YpPCP1.03 | 9.4 |
|  | | | | |
| **B. Chromosomal genes** | | | | |
| *Amino acid transport and metabolism* | | | | |
| *asnA* | y0003 | asparagine synthetase AsnA | YPO0003 | *b* |
| *metA* | y0013 | homoserine O-succinyltransferase | YPO3727 | 8.8 |
| *lysC* | y0023 | aspartate kinase III | YPO3719 | *b* |
| *metL* | y0303 | bifunctional aspartate kinase II/homoserine dehydrogenase II | YPO0116 | 7.6 |
| *metF* | y0304 | 5,10-methylenetetrahydrofolate reductase | YPO0117 | 32.2 |
| *argC* | y0310 | N-acetyl-gamma-glutamyl-phosphate reductase | YPO3927 | *b* |
| *ilvC* | y0345 | ketol-acid reductoisomerase | YPO3888 | 5.2 |
| *livM* | y0424 | leucine/isoleucine/valine transporter permease subunit | YPO3806 | *b* |
| *metE* | y0442 | 5-methyltetrahydropteroyltriglutamate--homocysteine methyltransferase | YPO3788 | *b* |
| *metC* | y0538 | cystathionine beta-lyase (beta-cystathionase) | YPO0278 | *b* |
| *-* | y0939 | N-carbamoyl-L-amino acid amidohydrolase | YPO3249 | *b* |
| *ureA* | y1237 | urease subunit gamma | YPO2665 | 16.6 |
| *ureB* | y1238 | urease subunit beta | YPO2666 | 8.9 |
| *ureC* | y1239 | urease subunit alpha | YPO2667 | 7.5 |
| *ureG* | y1242 | urease accessory protein | YPO2670 | *b* |
| *glyA* | y1322 | serine hydroxymethyltransferase | YPO2907 | 7.0 |
| *pepB* | y1342 | aminopeptidase B | YPO2889 | *b* |
| *nanA* | y1458 | acetylneuraminate lyase | YPO3024 | *b* |
| *-* | y1461 | N-acetylmannosamine kinase | YPO3020 | *b* |
| *cysK* | y1489 | cysteine synthase A | YPO2992 | 4.8 |
| *-* | y1507 | aminotransferase | YPO2975 | 4.0 |
| *mnmC* | y1590 | 5-methylaminomethyl-2-thiouridine methyltransferase | YPO2756 | 4.3 |
| *mppA* | y1993 | transport periplasmic protein | YPO2339 | 4.9 |
| *ybtS* | y2394 | salicylate synthase Irp9 | YPO1916 | 84.0 |
| *hisD* | y2621 | histidinol dehydrogenase | YPO1548 | *b* |
| *hisB* | y2623 | imidazole glycerol-phosphate dehydratase/histidinol phosphatase | YPO1546 | 5.2 |
| *ysuJ* | y2641 | decarboxylase | YPO1529 | 5.6 |
| *aspC* | y2760 | aromatic amino acid aminotransferase | YPO1410 | 7.0 |
| *-* | y2828 | chorismate mutase | YPO1353 | 4.2 |
| *potH* | y2848 | putrescine transporter subunit: membrane component of ABC superfamily | YPO1333 | *b* |
| *-* | y2870 | putative hydrolase | YPO1315 | *b* |
| *adiA* | y2987 | biodegradative arginine decarboxylase | YPO1201 | *b* |
| *serA* | y3301 | D-3-phosphoglycerate dehydrogenase | YPO0914 | 4.3 |
| *proC* | y3328 | pyrroline-5-carboxylate reductase | YPO0942 | 4.5 |
| *speC* | y3347 | ornithine decarboxylase isozyme | YPO0960 | *b* |
| *metC* | y3496 | cystathionine beta-lyase | YPO0681 | *b* |
| *leuB* | y3646 | 3-isopropylmalate dehydrogenase | YPO0532 | *b* |
| *leuC* | y3647 | isopropylmalate isomerase large subunit | YPO0531 | *b* |
| *dapB* | y3694 | dihydrodipicolinate reductase | YPO0480 | *b* |
| *thrA* | y3718 | bifunctional aspartokinase I/homeserine dehydrogenase I | YPO0459 | *b* |
| *glnA* | y3804 | glutamine synthetase | YPO0024 | 4.2 |
| *prlC* | y3855 | oligopeptidase A | YPO3975 | 4.7 |
| *tdcB* | y4105 | hypothetical protein | YPO4089 | 5.2 |
| *-* | y4106 | ornithine cyclodeaminase | YPO4090 | *b* |
|  | | | | |
| *Carbohydrate transport and metabolism* | | | | |
| *pgi* | y0024 | glucose-6-phosphate isomerase | YPO3718 | 12.3 |
| *tpiA* | y0052 | triosephosphate isomerase | YPO0085 | 8.1 |
| *-* | y0248 | ribokinase | YPO3621 | *b* |
| *eno* | y0814 | phosphopyruvate hydratase | YPO3376 | 13.9 |
| *sgbU* | y0856 | putative L-xylulose 5-phosphate 3-epimerase | YPO3333 | *b* |
| *cpsG* | y1083 | phosphomannomutase | YPO3097 | *b* |
| *nagA* | y1201 | N-acetylglucosamine-6-phosphate deacetylase | YPO2626 | 4.6 |
| *nagB* | y1202 | glucosamine-6-phosphate deaminase | YPO2627 | 4.5 |
| *gntV* | y1646 | gluconokinase 1 | YPO2540 | *b* |
| *-* | y1687 | solute binding protein of ABC transporter | YPO2501 | *b* |
| *cpsG* | y1710 | phosphomannomutase | YPO2479 | *b* |
| *togB* | y1881 | periplasmic binding protein of oligogalacturonide ABC transporter | YPO1719 | *b* |
| *togA* | y1882 | ABC transporter, ATP-binding protein | YPO1720 | *b* |
| *-* | y1948 | inner membrane transport protein YdhC | YPO2389 | *b* |
| *-* | y2008 | D-arabinitol dehydrogenase | YPO2325 | *b* |
| *-* | y2108 | transport protein | YPO2266 | *b* |
| *gapA* | y2165 | glyceraldehyde-3-phosphate dehydrogenase | YPO2157 | 11.1 |
| *eda* | y2243 | keto-hydroxyglutarate-aldolase/keto-deoxy-phosphogluconate aldolase | YPO2067 | *b* |
| *zwf* | y2244 | glucose-6-phosphate 1-dehydrogenase | YPO2066 | 23.9 |
| *pykA* | y2246 | pyruvate kinase | YPO2064 | 4.7 |
| *-* | y2319 | putative ribose 5-phosphate isomerase | YPO1991 | *b* |
| *ybtX* | y2395 | hypothetical protein | YPO1915 | 19.1 |
| *malK* | y2414 | ATP-binding component of transport system for maltose | YPO1896 | *b* |
| *-* | y2491 | hypothetical protein | YPO1816 | *b* |
| *manX* | y2551 | PTS enzyme IIAB, mannose-specific | YPO1758 | 12.6 |
| *manY* | y2552 | PTS enzyme IIC, mannose-specific | YPO1757 | 6.1 |
| *manZ* | y2553 | mannose-specific PTS system protein IID | YPO1756 | 5.7 |
| *-* | y2585 | periplasmic binding protein of ABC transporter | YPO1579 | *b* |
| *gnd* | y2629 | 6-phosphogluconate dehydrogenase | YPO1541 | 4.5 |
| *-* | y2651 | ABC transporter binding protein | YPO1517 | 4.7 |
| *fruA* | y2885 | fructose-specific PTS system IIBC component | YPO1300 | *b* |
| *fruK* | y2886 | 1-phosphofructokinase | YPO1299 | *b* |
| *fruB* | y2887 | fructose-specific PTS IIA/HPr components | YPO1298 | *b* |
| *bglA* | y2932 | 6-phospho-beta-glucosidase A | YPO1254 | 18.2 |
| *galK* | y3045 | galactokinase | YPO1137 | 5.5 |
| *gpmA* | y3048 | phosphoglyceromutase | YPO1133 | 18.7 |
| *agaV* | y3219 | N-acetylgalactosamine PTS family enzyme IIB component | YPO0834 | *b* |
| *-* | y3220 | N-acetylgalactosamine PTS system enzyme IIC component | YPO0835 | *b* |
| *agaD* | y3221 | PTS permease protein | YPO0836 | *b* |
| *-* | y3223 | GlcNAc 6-P deacetylase | YPO0838 | *b* |
| *-* | y3238 | putative galactosidase n | YPO0853 | *b* |
| *malG* | y3239 | malG type permease | YPO0854 | *b* |
| *malF* | y3240 | malF-type permease | YPO0855 | 4.4 |
| *fba* | y3307 | fructose-bisphosphate aldolase | YPO0920 | 4.9 |
| *pgk* | y3308 | phosphoglycerate kinase | YPO0921 | 4.4 |
| *tktA* | y3310 | transketolase | YPO0926 | 6.6 |
| *-* | y3562 | glycosidase | YPO0616 | *b* |
| *-* | y3568 | hypothetical protein | YPO0610 | *b* |
| *uxaA* | y3598 | altronate hydrolase | YPO0581 | *b* |
| *uxaB* | y3599 | altronate oxidoreductase | YPO0580 | *b* |
| *uxaC* | y3600 | glucuronate isomerase | YPO0579 | *b* |
| *talB* | y3712 | transaldolase B | YPO0463 | 4.7 |
| *deoB* | y3741 | phosphopentomutase | YPO0439 | 4.6 |
| *gntV* | y3876 | gluconokinase 1 | YPO3953 | *b* |
| *glgX* | y3887 | glycogen debranching enzyme | YPO3941 | 4.2 |
| *glgC* | y3888 | glucose-1-phosphate adenylyltransferase | YPO3940 | 5.3 |
| *glgA* | y3889 | glycogen synthase | YPO3939 | 4.8 |
| *glgP* | y3890 | glycogen phosphorylase | YPO3938 | 11.5 |
|  | | | | |
| *Cell cycle control, mitosis and meiosis* | | | | |
| *-* | y0652 | cell morphogenesis/cell wall metabolism regulator | YPO3531 | 6.3 |
| *minD* | y2233 | cell division inhibitor MinD | YPO2077 | 4.2 |
|  | | | | |
| *Cell motility* | | | | |
| *cheZ* | y1843 | chemotaxis regulator CheZ | YPO1681 | *b* |
| *-* | y1858 | hypothetical fimbrial usher protein | YPO1696 | *b* |
| *flgG* | y2505 | cell-distal portion of basal-body rod | YPO1804 | *b* |
| *-* | y3441 | lateral flagellin | YPO0737 | *b* |
| *flgK* | y3446 | flagellar hook-associated protein FlgK | YPO0732 | *b* |
| *flgI* | y3448 | flagellar basal body P-ring protein | YPO0730 | *b* |
| *flgH* | y3449 | flagellar basal body L-ring protein | YPO0729 | *b* |
| *flgG* | y3450 | flagellar basal body rod protein FlgG | YPO0728 | *b* |
| *fliH* | y3462 | flagellar assembly protein H | YPO0716 | *b* |
| *fliG* | y3463 | flagellar motor switch protein G | YPO0715 | *b* |
| *-* | y3465 | hypothetical protein | YPO0713 | *b* |
| *-* | y3479 | chaperone | YPO0699 | 4.3 |
|  | | | | |
| *Cell wall/membrane biogenesis* | | | | |
| *hasB* | y0317 | TonB-like protein | YPO3918 | *b* |
| *-* | y0545 | orfY protein in hemin uptake locus | YPO0284 | 11.9 |
| *acrA* | y1050 | acridine efflux pump | YPO3132 | 5.5 |
| *nlpB* | y1419 | lipoprotein | YPO3061 | 4.9 |
| *-* | y1530 | hypothetical OmpA-family protein in fimbrial locus | YPO2953 | *b* |
| *slyB* | y1962 | outer membrane protein | YPO2373 | 4.6 |
| *-* | y2020 | component of insecticidal toxin complex | YPO2312 | 10.1 |
| *tonB* | y2037 | transport protein TonB | YPO2193 | 7.0 |
| *ompC* | y2966 | outer membrane porin protein C | YPO1222 | 5.5 |
| *galE* | y3043 | UDP-galactose-4-epimerase | YPO1139 | 5.2 |
| *galT* | y3044 | galactose-1-phosphate uridylyltransferase | YPO1138 | 5.6 |
| *flgJ* | y3447 | peptidoglycan hydrolase | YPO0731 | *b* |
| *slt* | y3727 | lytic murein transglycosylase | YPO0452 | 4.4 |
|  | | | | |
| *Coenzyme transport and metabolism* | | | | |
| *-* | y0055 | hypothetical protein | YPO0082 | *b* |
| *panF* | y0211 | sodium/panthothenate symporter | YPO3657 | *b* |
| *btuB* | y0325 | vitamin B12/cobalamin outer membrane transporter | YPO3910 | 4.2 |
| *hemX* | y0379 | putative uroporphyrinogen III C-methyltransferase | YPO3851 | 6.0 |
| *thiC* | y0491 | thiamine biosynthesis protein ThiC | YPO3739 | *b* |
| *-* | y0547 | coproporphyrinogen III oxidase | YPO0286 | 5.1 |
| *panC* | y0785 | pantoate-beta-alanine ligase | YPO3402 | 7.3 |
| *pdxJ* | y1300 | pyridoxal phosphate biosynthetic protein PdxJ | YPO2930 | 4.0 |
| *bioA* | y3032 | adenosylmethionine--8-amino-7-oxononanoate transaminase | YPO1150 | *b* |
| *metK* | y3314 | S-adenosylmethionine synthetase | YPO0931 | 16.1 |
| *-* | y3332 | coproporphyrinogen III oxidase | YPO0946 | *b* |
| *tbpA* | y3651 | thiamin transporter substrate binding subunit | YPO0522 | *B* |
| *cysG* | y3941 | siroheme synthase | YPO0158 | 7.7 |
|  | | | | |
| *Defense mechanisms* | | | | |
| *-* | y1845 | N-acetylmuramoyl-L-alanine amidase | YPO1683 | 6.4 |
| *ybtQ* | y2396 | permease/ATP-binding protein of yersiniabactin-iron ABC transporter | YPO1914 | 17.4 |
| *ybtP* | y2397 | permease/ATP-binding protein of yersiniabactin-iron ABC transporter | YPO1913 | 15.0 |
|  | | | | |
| *Energy production and conversion* | | | | |
| *ppc* | y0308 | phosphoenolpyruvate carboxylase | YPO3929 | 5.6 |
| *qor* | y0576 | quinone oxidoreductase, NADPH-dependent | YPO0319 | *b* |
| *hydN* | y0600 | electron transport protein | YPO0343 | *B* |
| *aceE* | y0767 | pyruvate dehydrogenase subunit E1 | YPO3419 | 16.4 |
| *aceF* | y0768 | dihydrolipoamide acetyltransferase | YPO3418 | 11.9 |
| *lpdA* | y0769 | dihydrolipoamide dehydrogenase | YPO3417 | 6.9 |
| *-* | y0958 | hypothetical protein | YPO3232 | *b* |
| *hmp* | y1321 | flavohemoglobin nitric oxide dioxygenase | YPO2908 | *b* |
| *maeB* | y1449 | malic enzyme | YPO3034 | 5.0 |
| *ldhA* | y2004 | D-lactate dehydrogenase | YPO2329 | 6.3 |
| *adhE* | y2023 | CoA-linked acetaldehyde dehydrogenase | YPO2180 | 24.8 |
| *fumC* | y2106 | fumarate hydratase | YPO2264 | 6.2 |
| *pntA* | y2135 | NAD(P) transhydrogenase subunit alpha | YPO2303 | 7.0 |
| *ripA* | y2385 | Coenzyme A transferase | YPO1926 | *b* |
| *putA* | y2455 | trifunctional transcriptional regulator/proline dehydrogenase/pyrroline-5-carboxylate dehydrogenase | YPO1851 | 8.9 |
| *pflB* | y2790 | formate acetyltransferase 1 | YPO1383 | 8.2 |
| *-* | y2820 | HCP oxidoreductase, NADH-dependent | YPO1359 | *b* |
| *-* | y3177 | hypothetical aldo-keto reductase tas | YPO0790 | 4.7 |
| *nirB* | y3945 | nitrite reductase (NAD(P)H) subunit | YPO0161 | *b* |
| *atpF* | y4139 | F0F1 ATP synthase subunit B | YPO4125 | 7.9 |
|  | | | | |
| *Inorganic ion transport and metabolism* | | | | |
| *zntA* | y0410 | zinc/cadmium/mercury/lead-transporting ATPase | YPO3820 | *b* |
| *hmuT* | y0541 | periplasmic heme binding protein | YPO0281 | *b* |
| *hmuS* | y0542 | heme uptake system component | YPO0282 | 37.1 |
| *hmuR* | y0543 | TonB-dependent outer membrane receptor | YPO0283 | *b* |
| *-* | y0546 | orfX protein in heme uptake locus | YPO0285 | 7.9 |
| *terB* | y0557 | tellurium resistance protein | YPO0296 | 4.5 |
| *cysC* | y0825 | adenylylsulfate kinase | YPO3364 | *b* |
| *katG(Y)* | y0870 | catalase; hydroperoxidase HPI(I) | YPO3319 | 5.5 |
| *kdpA* | y1265 | potassium-transporting ATPase subunit A | YPO2692 | *b* |
| *pstB* | y1401 | phosphate-specific transport component | YPO2833 | *b* |
| *-* | y1457 | hypothetical protein | YPO3025 | 4.7 |
| *cysA* | y1469 | sulfate/thiosulfate transporter subunit | YPO3012 | *b* |
| *yfuA* | y1526 | solute-binding periplasmic protein for iron ABC transporter | YPO2958 | 9.5 |
| *yfeB* | y1896 | ATP-binding protein for iron and manganese ABC transporter | YPO2440 | 6.7 |
| *yfeA* | y1897 | periplasmic-binding protein for iron and manganese ABC transporter | YPO2439 | 14.4 |
| *psn* | y2404 | pesticin/yersiniabactin outer membrane receptor | YPO1906 | 32.3 |
| *ysuR* | y2633 | outer membrane iron/siderophore receptor | YPO1537 | *b* |
| *ysuA* | y2634 | solute-binding periplasmic protein of ABC transporter | YPO1536 | *b* |
| *ysuD* | y2637 | ATP-binding component for iron transport system | YPO1533 | *b* |
| *ysuG* | y2638 | siderophore biosynthetic protein | YPO1532 | *b* |
| *ysuI* | y2640 | siderophore biosynthetic protein | YPO1530 | *b* |
| *yiuR* | y2872 | outer membrane iron/siderophore receptor | YPO1313 | *b* |
| *yiuC* | y2873 | ATP-binding protein of iron/siderophore ABC transporter | YPO1312 | 6.4 |
| *yiuA* | y2875 | solute-binding periplasmic protein of iron/siderophore ABC transporter | YPO1310 | 4.1 |
| *metN* | y3104 | DL-methionine transporter ATP-binding subunit | YPO1073 | 4.1 |
| *-* | y3105 | DL-methionine transporter permease subunit | YPO1072 | 6.0 |
| *metQ* | y3106 | DL-methionine transporter substrate-binding subunit | YPO1071 | 14.0 |
| *-* | y3343 | TonB-dependent outer membrane receptor | YPO0956 | 4.2 |
| *-* | y3404 | OM receptor in Ynp siderophore locus | YPO1011 | *b* |
| *nirC* | y3942 | nitrite transporter NirC | YPO0159 | *b* |
| *nirD* | y3944 | nitrite reductase small subunit | YPO0160 | 7.3 |
| *-* | y4043 | solute-binding iron ABC transport protein | YPO4022 | 4.4 |
| *sodA* | y4080 | superoxide dismutase | YPO4061 | 4.7 |
|  | | | | |
| *Intracellular trafficking and secretion* | | | | |
| *ftsY* | y0416 | cell division membrane protein | YPO3814 | *b* |
| *exbD* | y3494 | biopolymer transport protein ExbD | YPO0683 | 7.5 |
| *exbB* | y3495 | TonB complex protein | YPO0682 | 10.0 |
| *-* | y3579 | filamentous hemagglutinin | YPO0599 | 6.7 |
| *-* | y3824 | hemolysin activator protein precursor | YPO4005 | *b* |
|  | | | | |
|  | | | | |
| *Lipid transport and metabolism* | | | | |
| *accC* | y0209 | acetyl-CoA carboxylase biotin carboxylase subunit | YPO3658 | 7.2 |
| *-* | y0219 | oxidoreductase | YPO3648 | 5.5 |
| *fadB* | y0464 | multifunctional fatty acid oxidation complex subunit alpha | YPO3766 | 6.2 |
| *acpD* | y2010 | acyl carrier protein phosphodiesterase | YPO2323 | 4.4 |
| *cdh* | y3843 | CDP-diacylglycerol pyrophosphatase | YPO3986 | 4.0 |
| *-* | y4042 | hypothetical protein | YPO4021 | 4.0 |
|  | | | | |
| *Nucleotide transport and metabolism* | | | | |
| *pyrB* | y0161 | aspartate carbamoyltransferase catalytic subunit | YPO3588 | *b* |
| *pyrI* | y0162 | aspartate carbamoyltransferase regulatory subunit | YPO3589 | 8.4 |
| *udp* | y0444 | uridine phosphorylase | YPO3786 | 5.4 |
| *purD* | y0501 | phosphoribosylamine--glycine ligase | YPO3729 | *b* |
| *purH* | y0502 | bifunctional phosphoribosylaminoimidazolecarboxamide formyltransferase/IMP cyclohydrolase | YPO3728 | *b* |
| *cpdB* | y0653 | bifunctional 2',3'-cyclic nucleotide 2'-phosphodiesterase/3'-nucleotidase periplasmic precursor protein | YPO3530 | *b* |
| *nrdF* | y1222 | ribonucleotide-diphosphate reductase subunit beta | YPO2648 | *b* |
| *nrdE* | y1223 | ribonucleotide-diphosphate reductase subunit alpha | YPO2649 | *b* |
| *nrdH* | y1225 | glutaredoxin-like protein | YPO2651 | *b* |
| *purL* | y1309 | phosphoribosylformylglycinamidine synthase | YPO2921 | 4.1 |
| *upp* | y1408 | uracil phosphoribosyltransferase | YPO2827 | 5.5 |
| *deoC* | y2860 | deoxyribose-phosphate aldolase | YPO1323 | 4.7 |
| *-* | y3744 | transport system permease protein | YPO0435 | *b* |
| *codA* | y3946 | cytosine deaminase | YPO0162 | *b* |
|  | | | | |
| *Posttranslational modification, protein turnover, chaperones* | | | | |
| *hslU* | y0294 | ATP-dependent protease ATP-binding subunit | YPO0105 | 6.8 |
| *groES* | y0608 | co-chaperonin GroES | YPO0350 | 11.4 |
| *groEL* | y0609 | chaperonin GroEL | YPO0351 | 16.1 |
| *msrA* | y0658 | methionine sulfoxide reductase A | YPO3525 | 8.5 |
| *clpB* | y0914 | protein disaggregation chaperone | YPO3275 | 8.2 |
| *-* | y0988 | peroxidase | YPO3194 | 7.4 |
| *tig* | y1026 | trigger factor | YPO3158 | 8.3 |
| *-* | y1031 | peptidyl-prolyl cis-trans isomerase (rotamase D) | YPO3153 | 4.2 |
| *htpG* | y1064 | heat shock protein 90 | YPO3119 | 5.6 |
| *-* | y1097 | thioredoxin-like protein | YPO3082 | 8.1 |
| *sufB* | y1935 | cysteine desulfurase activator complex subunit | YPO2403 | 4.2 |
| *gst* | y1968 | glutathionine S-transferase | YPO2367 | 7.2 |
| *tpx* | y1990 | thiol peroxidase | YPO2342 | 5.4 |
| *trxB* | y2802 | thioredoxin reductase | YPO1374 | 4.0 |
| *clpA* | y2809 | ATP-dependent Clp protease ATP-binding subunit | YPO1368 | 5.8 |
| *-* | y3669 | ATP-dependent protease | YPO0506 | *b* |
|  | | | | |
| *Replication, recombination and repair* | | | | |
| *mutM* | y0090 | formamidopyrimidine-DNA glycosylase | YPO0052 | *b* |
| *dnaB* | y0577 | replicative DNA helicase | YPO0320 | 4.8 |
| *deaD* | y0696 | ATP-dependent RNA helicase DeaD | YPO3488 | 10.1 |
| *sbcD* | y0977 | exonuclease subunit SbcD | YPO3206 | *b* |
| *hupB* | y1030 | transcriptional regulator HU subunit beta | YPO3154 | 4.0 |
| *priC* | y1056 | primosomal replication protein N | YPO3127 | *b* |
| *xthA* | y2153 | exonuclease III | YPO2168 | *b* |
| *mutY* | y3339 | adenine DNA glycosylase | YPO0952 | *b* |
| *-* | y3540 | hypothetical protein | YPO0641a | *b* |
|  | | | | |
| *Secondary metabolites biosynthesis, transport and catabolism* | | | | |
| *-* | y0777 | multicopper oxidase | YPO3409 | *b* |
| *-* | y0984 | short chain dehydrogenase | YPO3199 | 6.5 |
| *-* | y2318 | hypothetical protein | YPO1992 | 4.2 |
| *irp2* | y2399 | HMWP2 nonribosomal peptide synthetase (yersiniabactin) | YPO1911 | 38.0 |
| *irp1* | y2400 | HMWP1 nonribosomal peptide/polyketide synthase (yersiniabactin) | YPO1910 | 46.7 |
| *ybtU* | y2401 | thiazolinyl-S-HMWP1 reductase | YPO1909 | 27.0 |
| *ybtE* | y2403 | salicyl-AMP ligase | YPO1907 | 26.8 |
| *fabG* | y2717 | 2,5-dichloro-2,5-cyclohexadiene-1,4-diol dehydrogenase | YPO1452 | *b* |
| *-* | y2836 | ABC transport protein | YPO1346 | *b* |
| *tauD* | y3966 | taurine dioxygenase | YPO0185 | *b* |
|  | | | | |
| *Signal transduction mechanisms and transcription* | | | | |
| *-* | y0053 | transcriptional regulator | YPO0084 | *b* |
| *treR* | y0165 | trehalose repressor | YPO3698 | *b* |
| *cytR* | y0297 | DNA-binding transcriptional regulator | YPO0108 | 4.0 |
| *-* | y0390 | transcriptional regulator | YPO3840 | 4.2 |
| *metR* | y0441 | regulator for metE and metH | YPO3789 | 9.4 |
| *rpoB* | y0484 | DNA-directed RNA polymerase subunit beta | YPO3747 | 6.6 |
| *pmrB* | y0676 | sensor protein BasS/PmrB | YPO3508 | *b* |
| *-* | y1094 | MerR family transcriptional regulator | YPO3085 | 4.5 |
| *-* | y1731 | hypothetical protein | YPO2458 | *B* |
| *rovA* | y1961 | transcriptional regulator SlyA | YPO2374 | 8.7 |
| *-* | y2012 | hypothetical protein | YPO2320 | *b* |
| *-* | y2191 | phage antirepressor | YPO2126 | *b* |
| *HexR* | y2245 | DNA-binding transcriptional regulator | YPO2065 | 8.2 |
| *ybtA* | y2398 | AraC-type transcriptional regulator for yersiniabactin uptake and biosynthetic genes | YPO1912 | *b* |
| *-* | y2419 | transcriptional regulator of the GntR family | YPO1890 | *b* |
| *flgM* | y2513 | anti-sigma28 factor FlgM | YPO1797 | *b* |
| *psaE* | y2884 | regulator | YPO1301 | 12.1 |
| *-* | y2919 | ATP-dependent helicase | YPO1265 | *b* |
| *rpoD* | y3537 | RNA polymerase sigma factor RpoD | YPO0643 | 4.7 |
| *hepA* | y3656 | ATP-dependent helicase HepA | YPO0517 | *b* |
|  | | | | |
| *Translation* | | | | |
| *-* | y0071 | hypothetical protein | YPO0071 | *b* |
| *rph* | y0097 | ribonuclease PH | YPO0044 | *b* |
| *-* | y0163 | hypothetical protein | YPO3590 | 4.6 |
| *trmA* | y0324 | tRNA (uracil-5-)-methyltransferase | YPO3911 | *b* |
| *rplK* | y0480 | 50S ribosomal protein L11 | YPO3751 | 4.2 |
| *rpsF* | y0645 | 30S ribosomal protein S6 | YPO3539 | 6.7 |
| *infB* | y0688 | translation initiation factor IF-2 | YPO3496 | 6.3 |
| *-* | y0910 | yhbH sigma 54 modulator | YPO3279 | 8.2 |
| *rpmE2* | y1048 | 50S ribosomal protein L31 type B | YPO3134 | *b* |
| *-* | y1195 | hypothetical protein | YPO2620 | 4.9 |
| *pheS* | y1908 | phenylalanyl-tRNA synthetase subunit alpha | YPO2429 | 4.3 |
| *pheT* | y1909 | phenylalanyl-tRNA synthetase subunit beta | YPO2428 | 4.1 |
| *-* | y2070 | translation initiation factor Sui1 | YPO2228 | 5.1 |
| *tuf* | y3986 | elongation factor Tu | YPO0203 | 4.9 |
| *rpsJ* | y3989 | 30S ribosomal protein S10 | YPO0209 | 7.9 |
| *rplD* | y3991 | 50S ribosomal protein L4 | YPO0211 | 7.7 |
| *rplW* | y3992 | 50S ribosomal protein L23 | YPO0212 | 7.5 |
| *rplB* | y3993 | 50S ribosomal protein L2 | YPO0213 | 4.6 |
| *rpsC* | y3995 | 30S ribosomal protein S3 | YPO0216 | 16.4 |
| *rplP* | y3996 | 50S ribosomal protein L16 | YPO0217 | 6.9 |
| *rpmC* | y3998 | 50S ribosomal protein L29 | YPO0218 | *b* |
| *rplN* | y3999 | 50S ribosomal protein L14 | YPO0220 | 5.8 |
| *rpsE* | y4007 | 30S ribosomal protein S5 | YPO0226 | 4.0 |
| *rplQ* | y4016 | 50S ribosomal protein L17 | YPO0235 | 4.0 |
|  | | | | |
| *General function and prediction and function unknown* | | | | |
| *-* | y0036 | hemolysin co-regulated protein | YPO3708 | *b* |
| *-* | y0038 | hypothetical protein in putative T6SS (IAHP) locus | YPO3706 | *b* |
| *-* | y0039 | hypothetical protein in putative T6SS (IAHP) locus | YPO3705 | *b* |
| *-* | y0054 | hypothetical protein | YPO0083 | *b* |
| *-* | y0123 | conserved exported protein | YPO3551 | 5.4 |
| *-* | y0152 | hypothetical protein | YPO3580 | 5.9 |
| *-* | y0205 | putative sulfite oxidase subunit YedY | YPO3662 | 7.1 |
| *-* | y0210 | hypothetical protein | YPO3657a | *b* |
| *-* | y0218 | decarboxylase | YPO3649 | 5.5 |
| *-* | y0741 | hypothetical protein | YPO3445 | 4.6 |
| *-* | y0821 | hypothetical protein | YPO3369 | 6.0 |
| *-* | y0962 | hypothetical protein | YPO3227 | *b* |
| *-* | y1035 | hypothetical protein | YPO3149 | *b* |
| *-* | y1125 | hypothetical protein | YPO2805 | *b* |
| *-* | y1276 | hypothetical protein | YPO2700 | *b* |
| *-* | y1282 | formate acetyltransferase | YPO2705 | 8.5 |
| *yplB* | y1285 | accessory protein for YplA | YPO2707 | *b* |
| *-* | y1399 | inner membrane permease of high-affinity phosphate-specific transport system | YPO2835 | *b* |
| *-* | y1455 | hypothetical protein | YPO3027 | 5.0 |
| *-* | y1516 | oxidoreductase component | YPO2968 | *b* |
| *-* | y1545 | hypothetical protein in putative T6SS (IAHP) locus | YPO2939 | *b* |
| *-* | y1546 | hypothetical protein in putative T6SS (IAHP) locus | YPO2938 | *b* |
| *hns* | y2146 | global DNA-binding transcriptional dual regulator | YPO2175 | 6.9 |
| *mviM* | y2270 | virulence factor | YPO2042 | 8.2 |
| *-* | y2448 | hypothetical protein | YPO1858 | *b* |
| *-* | y2562 | hypothetical protein | YPO1747 | *b* |
| *-* | y2589 | nucleoprotein/polynucleotide-associated enzyme | YPO1575 | 4.7 |
| *-* | y2652 | hypothetical protein | YPO1516 | 4.5 |
| *-* | y2687 | hypothetical protein in putative T6SS (IAHP) locus | YPO1483 | *b* |
|  | y2698 | hypothetical protein in putative T6SS (IAHP) locus | YPO1472 |  |
| *-* | y2700 | hypothetical Hcp-like protein in putative T6SS (IAHP) locus | YPO1470 | *b* |
| *-* | y2906 | hypothetical protein | YPO1277 | 4.6 |
| *-* | y3087 | hypothetical protein | YPO1090 | *b* |
| *dkgB* | y3101 | 2,5-diketo-D-gluconate reductase B | YPO1075 | *b* |
| *-* | y3327 | hypothetical protein | YPO0941 | 4.2 |
| *-* | y3594 | hypothetical protein | YPO0585 | 4.0 |
| *-* | y3650 | solute-binding periplasmic protein of ABC transporter | YPO0524 | *b* |
| *-* | y3659 | hypothetical protein | YPO0514 | *b* |
| *-* | y3837 | hypothetical protein | YPO3991 | 6.6 |
| *-* | y3872 | hypothetical protein | YPO3956 | *b* |
| *-* | y4109 | predicted hydrolase | YPO4093 | *b* |
| *trmE* | y4118 | tRNA modification GTPase TrmE | YPO4103 | 5.3 |
|  | | | | |
| *Not in COGs* | | | | |
| *-* | y0164 | hypothetical protein | YPO3699 | 8.6 |
| *-* | y0237 | hypothetical protein | YPO3631 | *b* |
| *-* | y0256 | hypothetical protein | YPO3614 | *b* |
| *-* | y0391 | hypothetical protein | YPO3839 | 8.3 |
| *-* | y0392 | hypothetical protein | YPO3838 | 13.9 |
| *-* | y0433 | hypothetical protein | YPO3797 | *b* |
| *-* | y0552 | hypothetical protein | YPO0291 | *b* |
| *-* | y0553 | hypothetical protein | YPO0292 | *b* |
| *-* | y0596 | hypothetical protein | YPO0337 | 11.8 |
| *-* | y0853 | hypothetical protein | YPO3336 | 6.0 |
| *-* | y0944 | adhesin system protein | YPO3246 | *b* |
| *-* | y1022 | sugar hydrolase | YPO3163 | *b* |
| *asr* | y1226 | acid shock protein precursor | YPO2652 | *b* |
| *sseB* | y1343 | enhanced serine sensitivity | YPO2888 | 5.7 |
| *-* | y1376 | hypothetical protein | YPO2857 | 8.0 |
| *-* | y1506 | hypothetical protein | YPO2976 | 50.7 |
| *-* | y1559 | hypothetical protein in putative T6SS (IAHP) locus | YPO2726 | *b* |
| *-* | y1718 | hypothetical protein | YPO2471 | *b* |
| *-* | y1811 | hypothetical protein | YPO1649 | 24.5 |
| *-* | y1987 | hypothetical protein | YPO2345 | 4.3 |
| *asr* | y2014 | acid shock protein | YPO2318 | *b* |
| *-* | y2073 | hypothetical protein | YPO2231 | *b* |
| *tus* | y2107 | DNA replication terminus site-binding protein | YPO2265 | 4.6 |
| *-* | y2185 | phage tail protein | YPO2134 | *b* |
| *-* | y2203 | hypothetical protein | YPO2112 | *b* |
| *-* | y2315 | hypothetical protein | YPO1995 | 4.4 |
| *-* | y2407 | hypothetical protein | YPO1902 | 4.9 |
| *-* | y2456 | hypothetical protein | YPO1850 | 9.5 |
| *psaA* | y2882 | pH 6 Antigen fimbrial subunit | YPO1303 | *b* |
| *-* | y2953 | hypothetical protein | YPO1234 | *b* |
| *-* | y2956 | virulence protein | YPO1232 | *b* |
| *-* | y3246 | hypothetical protein | YPO0862 | *b* |
| *-* | y3418 | hypothetical protein, Ynp siderophore locus | YPO0775 | *b* |
| *-* | y3444 | hypothetical protein | YPO0734 | *b* |
| *-* | y3458 | putative flagellar regulatory protein | YPO0720 | 4.9 |
| *-* | y3544 | hypothetical protein | YPO0634 | 4.12 |
| *-* | y3586 | hypothetical protein | YPO0593 | *b* |
| *-* | y4083 | hypothetical protein | YPO4064 | 13.3 |
| *-* | y4088 | hypothetical protein | YPO4070 | 4.6 |
| **b,* gene transcripts detected in the bubo only | | | | |
