# Supplementary material for: Transit through the Flea Vector Induces a Pretransmission Innate Immunity Resistance Phenotype in Yersinia pestis
Source: PLoS Pathog. 2010 Feb 26;6(2):e1000783. doi: 10.1371/journal.ppat.1000783 (PMC2829055; doi:10.1371/journal.ppat.1000783)
Supplement: Table S7 — Primers and probes used for quantitative RT-PCR. (0.06 MB DOC) [file ppat.1000783.s009.doc]

| **Table S7.** Primers and probes used for quantitative RT-PCR | | |
| --- | --- | --- |
| gene target | function | sequence (5’ – 3’) |
| *crr* | primers | GCC CTC TGG CAA TAA AAT GG (F); AGC ATG GTT GGT CTC GAA AAT T (R) |
|  | probe | CTC CTG TTG ACG GCA TCG GT |
| *potB* | primers | GAT GAG CGG CAG CAA TCT G (F); TGT GTA GGC CGC CTT CAC TT (R) |
|  | probe | CCC GAT ACT GCG TCA ACA ACT GCG |
| *potD* | primers | CGC ACA GGT TAA GGC GTT CT (F); CCC GGC TTC AAT TGT TCC T (R) |
|  | probe | ACC TTT CAT GGC CGC CGG |
| *gabT* | primers | CGC CAC CCG AAA GTG ATG (F); CAC CAC CTG ATA GGC AGT ATG G (R) |
|  | probe | CTG TGC GCC AAC AAC TGG ACC AGT |
| *y1861* | primers | GGG ATG ATG TGG TGG GTG TAT C (F); AAC CAG GCC ATA AAC CGG TAT (R) |
|  | probe | CAG GTT GCT ACC GGA CAA GTC GT |
| *y1862* | primers | GCC TGA GTA ATT TGG GAA CCA T (F); GCC ACT GAA TGC GAC GTT AAT (R) |
|  | probe | AAC TTC GGT AGT GTA GTG GCG CTA TCC TCAG |
| *y3170* | primers | TGC TGG CTT AAT TTG GGT AAG AC (F); GAA ATG ATG CTG GCG CTA AGT (R) |
|  | probe | CTG TGC TCA GCG CTA TCA GGC TAC CG |
| *glnA* | primers | GAT TCA ACA CTG ATT ATC CGT TGT G (F); ACG TGG ATC GCG GTC ATA G (R) |
|  | probe | CAT TCT TGA GCC AGG CAC CAT GCA G |
| *rovA* | primers | GCA CGA TTA GTT CGC GTT TG (F); TTT GAG TCA GTT CCA ACG GTT TC (R) |
|  | probe | CGC GCA TTA ATT GAC CAT CGG |
| *ureA* | primers | CAA GAG AAG TTG AAA AGC TCA TGA T (F); AAG CCA CGC GCT TTG C (R) |
|  | probe | ACA CGC TGT CTG ATG TGG CGT |
| *phoP* | primers | GAT GCG GCG GAA TAT TGG (F); ACG GCG TGA AAG GTC AAT CT (R) |
|  | probe | CCT CCC AAA TTA TTG AGC TAC CGC CCT |
| *phoQ* | primers | CCT GCA CCG CGC AAG T (F); CGC GGG AAC CGA ATG A (R) |
|  | probe | TGC GTT CCG AAC ATA ATG TTC TAG GAC GTG |
| *pmrA* | primers | cat gtc acc ctg cca gta ctc a (F); GCC CGT CAA CCC TGT CTT C (R) |
|  | probe | CCT CAC CGC ACG TGA TGC GC |
| *yitA* | primers | CCC TTG ATG TAG ATG CAG GAT TT (F); AAT GCC TTG CGG ATA ATC ACT T (R) |
|  | probe | CTT CCT TGA TCA GGG TTC GTG G |
| *yitB* | primers | CGG GCG CTG GAA GTA GAG (F); ATT TCT GCC AGA TTG AAC TTA TG (R) |
|  | probe | ACC GTT TCA TTG GCA CAA CAT TAT CAA CAG T |
| *yitC* | primers | CCT GAT CTT AGC TGG ACC CAA T (F); TGC GCT TGT GGA TGG AAA TAC (R) |
|  | probe | CCG TTA TCT ACC TTG CCA GCG G |
| *yipA* | primers | GCC GCT GGG TGG AG TAT G (F); GGC TTG CTC GTG GTA ACT TAG C (R) |
|  | probe | CCG GTG AAC ACA GTT TAG GCC GCC |
